# The *C*. *elegans* CHP1 homolog, *pbo-1*, functions in innate immunity by regulating the pH of the intestinal lumen

**DOI:** 10.1371/journal.ppat.1008134

**Published:** 2020-01-09

**Authors:** Saida Benomar, Patrick Lansdon, Aaron M. Bender, Blake R. Peterson, Josephine R. Chandler, Brian D. Ackley

**Affiliations:** 1 Department of Molecular Biosciences, The University of Kansas, Lawrence, KS, United States of America; 2 Department of Medicinal Chemistry, The University of Kansas, Lawrence, KS, United States of America; Stanford University, UNITED STATES

## Abstract

*Caenorhabditis elegans* are soil-dwelling nematodes and models for understanding innate immunity and infection. Previously, we developed a novel fluorescent dye (KR35) that accumulates in the intestine of *C*. *elegans* and reports a dynamic wave in intestinal pH associated with the defecation motor program. Here, we use KR35 to show that mutations in the Ca^2+^-binding protein, PBO-1, abrogate the pH wave, causing the anterior intestine to be constantly acidic. Surprisingly, *pbo-1* mutants were also more susceptible to infection by several bacterial pathogens. We could suppress pathogen susceptibility in *pbo-1* mutants by treating the animals with pH-buffering bicarbonate, suggesting the pathogen susceptibility is a function of the acidity of the intestinal pH. Furthermore, we use KR35 to show that upon infection by pathogens, the intestinal pH becomes neutral in a wild type, but less so in *pbo-1* mutants. *C*. *elegans* is known to increase production of reactive oxygen species (ROS), such as H_2_O_2,_ in response to pathogens, which is an important component of pathogen defense. We show that *pbo-1* mutants exhibited decreased H_2_O_2_ in response to pathogens, which could also be partially restored in *pbo-1* animals treated with bicarbonate. Ultimately, our results support a model whereby PBO-1 functions during infection to facilitate pH changes in the intestine that are protective to the host.

## Author summary

Innate immunity is critical for host defense against pathogens. However, questions remain about how the host senses and responds to pathogen invasion. Using a pH-sensitive fluorescent dye and a *Caenorhabditis elegans* pathogen infection model we show that pathogens induce changes in pH of the worm intestine. We also show that intestinal pH directly affects production of reactive oxygen species (e.g. H_2_O_2_) important for pathogen defense. Our results show that pH regulation is an important component of the innate immune response to pathogens.

## Introduction

As increased antibiotic resistance is being observed in clinical settings, bacterial infections are becoming a crisis-level global health burden. Host barriers to infection can be protective against dangerous infections in the absence of antibiotics, and new discoveries about the mechanisms of host protection might pave the way for new therapeutics to treat disease. Host barriers to infection can be physical (*e*.*g*. an exoskeleton or epidermal layer), chemical (*e*.*g*. shed-able mucosa that adhere to pathogens) or genetic (*e*.*g*. innate and/or adaptive immunity).

One particularly effective mechanism of host protection is the production of reactive oxygen species (ROS), *e*.*g*. superoxide (O_2_^–^) which can be converted to hydrogen peroxide (H_2_O_2_) [1]. Over the past 15–20 years H_2_O_2_ has emerged as a critical signaling molecule in several contexts, including infection and wound repair [2–4]. In the vertebrate innate immune response, the enzymes NADPH oxidase and superoxide dismutase (SOD) convert O_2_ to O_2_^-^ and H_2_O_2_ [5, 6]. H_2_O_2_ can be produced intracellularly and exported via aquaporin channels or produced in the extracellular space of cells responding to bacterial infection. Because ROS can also be self-destructive, it is no surprise that the production of ROS is tightly regulated in response to pathogens [7]. However, the identities of infection-dependent signals and regulatory responses that lead to ROS production remain incomplete.

Many discoveries about the role of ROS and other innate immune responses in organismal defenses to pathogens have come from genetically-tractable model systems where infection can be presented in a controlled manner. For example, the nematode *Caenorhabditis elegans* has served as an excellent system to understand the mechanisms that underlie organismal responses to pathogens and wounding [8–10]. Portions of the ROS production pathways are largely conserved in *C*. *elegans* and vertebrate animals, including humans. For example *itr-1*, the inositol trisphosphate receptor is required for intracellular calcium release in response to infection [1], an aquaporin, *aqp-1*, is required for the full anti-pathogen response [11], and a dual oxidase/peroxidase, *bli-3* and a secreted peroxidase, *skpo-1*, are important for the defense against pathogens [12, 13]. Importantly, these pathways are simplified in *C*. *elegans* with minimal redundancy. Taken together, the evolutionary conservation of organismal response to infection indicates that we can use *C*. *elegans* to better understand vertebrate response to bacterial pathogens.

This study is focused on PBO-1, a protein we find to be key in the response to pathogen infection. PBO-1 was first identified because of its contribution to the posterior body contraction (Pbo) phenotype [14]. PBO-1 is an EF-hand protein orthologous to human CHP1 (Calcineurin B homologous protein 1, also called P22) [15]. It is thought that PBO-1 links the release of intracellular calcium to the activity and/or trafficking of Na^+^/H^+^-exchangers (NHE or NHX), including NHX-2, NHX-6 and PBO-4 (also known as NHX-7) [16]. In *C*. *elegans*, PBO-4 is required for the release of protons from the basolateral membrane of the intestinal cell onto adjacent muscles, where they activate the proton-gated ion channel, PBO-5 [17].

In this study, we show that PBO-1 loss-of-function mutations fail to undergo oscillatory pH fluctuations in the intestine and have an intestinal pH that is more acidic than wild-type animals. Surprisingly, *pbo-1* mutants are more susceptible to pathogens than wild-type animals and show decreased production of ROS during infection. Our results also show that pathogen infections alter the intestinal pH in *C*. *elegans*, and that this regulation is at least partially dependent on PBO-1. We can suppress the acidic phenotype and pathogen susceptibility by removing PBO-4 from *pbo-1* mutant animals or by adding pH-neutralizing sodium bicarbonate to the *C*. *elegans* growth medium. Overall, we propose a model whereby infection by pathogens initiates a neutralization of the normally acidic pH in the intestine, which enables increased levels of ROS production to protect the host from the pathogen.

## Results

### *pbo-1* mutants have an abrogated pH wave in the intestine

**Fig 1:**
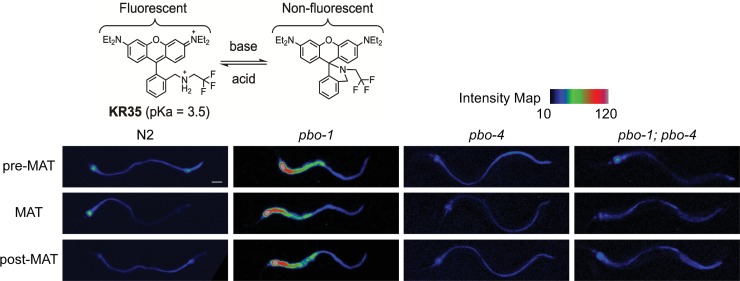
*pbo-1* mutations disrupt the intestinal acid wave. The chemical structures of KR35 under acidic and basic conditions are shown. The protonated ring-opened form is highly fluorescent, and the deprotonated spirocyclic form is non-fluorescent. The intensity map provides the lookup table values for the pixel intensity in each image. Fluorescence micrographs of *C*. *elegans* wild type or *pbo* mutants after feeding on the pH-sensitive probe KR35 for 30 min (10 μM). Time-dependent images during the DMP extracted from video microscopy are shown. Fluorescence is rendered as a heat map of fluorescence intensity with red representing the most intense fluorescence (high acidity), and black the least intense fluorescence (low acidity). The head of the animal is on the left side of each image (scale bar = 50 μm).

Previously we developed and characterized a pH-responsive dye, KR35, and used it to monitor pH in the intestine of *C*. *elegans* [18]. KR35 is activated by acid in a range that is physiologically relevant to the *C*. *elegans* intestine, which maintains a pH gradient from anterior to posterior of ~5.5–3.5 [19]. When we feed wild-type (N2) animals KR35 we observe stronger fluorescence in the posterior of the *C*. *elegans* intestine, indicating acidity in this region. We also observe a dynamic change in pH or an ‘acidic wave’, with a periodicity of ~50 seconds (Fig 1, Table 1, S1 Video, S1 Fig). During the oscillation, the posterior fluorescence transitions to the anterior region of the intestine near the pharyngeal-intestinal junction and stays there for about 3–7 seconds during a period we have termed the Maximum Anterior Transition (MAT). The 50-second oscillations and the MAT are coordinated with a process called the defecation motor program (DMP), whereby protons are removed from the posterior lumen of the intestine, then released at the basolateral membrane of the intestine by the Na^+^/H^+^ exchanger PBO-4, where they act as neurotransmitters that trigger muscle contractions immediately prior to the expulsion of waste [16, 17]. Using KR35 we previously showed that animals with mutations in *pbo-4* fail to produce the MAT during pH oscillations, although other aspects of the acidic wave were grossly normal such as the acidification of the posterior intestine prior to the MAT [18].

**Table 1:** MAT analysis using KR35.

Genotype	Bacteria	Animals with MATs (total observed)	MAT Interval (seconds)	MAT Average Fluorescence (AU)^a^
N2 (wild type)	*E*. *coli*	18 (18)	56 ± 6.1	12373.8 ± 8910.2
	*E*. *faecalis*	11 (16)	78 ± 3.1	8506.9 ± 6285.7
	*P*. *aeruginosa*	0 (20)	ND ^b^	2186.5 ± 1435.3
*pbo-1(sa7)*	*E*. *coli*	0 (15)	ND ^b^	22074.9 ± 13250.6
	*E*. *faecalis*	0 (16)	ND ^b^	16616.3 ± 9332.1
	*P*. *aeruginosa*	0 (16)	ND ^b^	7831.3 ± 6744.0
*pbo-4(ok583)*	*E*. *coli*	9 (15)	52 ± 1.1	4268.7 ± 2991.2
*pbo-1(sa7); pbo-4(ok583)*	*E*. *coli*	14 (15)	46 ± 0.4	4385.9 ± 3360.1

^a^ See S1 and S6 Figs for statistical analyses.

^b^ N.D. Not Determined (No MATs observed).

*pbo-4* mutants exhibit a Posterior body contraction absent phenotype (Pbo) because of a defect in contracting posterior muscles during DMP [17]. To evaluate the relationship between the MAT and Pbo phenotype, we examined another Pbo mutant, *pbo-1*(*sa7*). We found that *pbo-1* mutants fed KR35 failed to demonstrate any pH oscillations and instead had an anterior intestine exhibiting increased fluorescence relative to wild type, suggesting the region was continuously acidified (Fig 1, Table 1, S2 Video, S1 Fig). In contrast, the posterior intestine of the *pbo-1* mutant exhibited reduced KR35 fluorescence, relative to wild type (Fig 1). The reduced proton concentration in the posterior intestine likely explains the Pbo phenotype [17]. The anterior acidity in *pbo-1* mutants was the opposite of the *pbo-4* phenotype, where animals fail to acidify the anterior (Fig 1, Table 1, S3 Video, S1 Fig). Thus, we analyzed *pbo-1; pbo-4* double mutant animals using KR35. *pbo-1; pbo-4* double mutants had reduced anterior KR35 fluorescence (suggesting a more neutral intestinal pH) compared with *pbo-1* single mutants. Also, unlike *pbo-1* single mutants, the *pbo-1; pbo-4* double mutants exhibited pH oscillations, and protons were evacuated from the anterior during the MAT (Fig 1, Table 1, S4 Video, S1 Fig). Our observation that the *pbo-1; pbo-4* double mutants are more similar to the *pbo-4* single mutants suggests the increased acidity in the anterior intestine of the *pbo-1* mutants is dependent on *pbo-4* function.

Our results with KR35 are consistent with the known role of PBO-1 in trafficking PBO-4 [20], and more generally of the role of PBO-1-like molecules being regulators of NHX activity [21]. Our results would suggest that, at least in the anterior of the animal, PBO-1 can function as a negative regulator of PBO-4. In this case, loss of PBO-1 function would aberrantly activate PBO-4, leading to a PBO-4-dependent accumulation of protons in the anterior. Removing *pbo-4* from the *pbo-1* mutant obviates these effects, and somewhat normalizes the intestinal acidity (Fig 1).

### *pbo-1* mutants are more susceptible to pathogens

**Fig 2:**
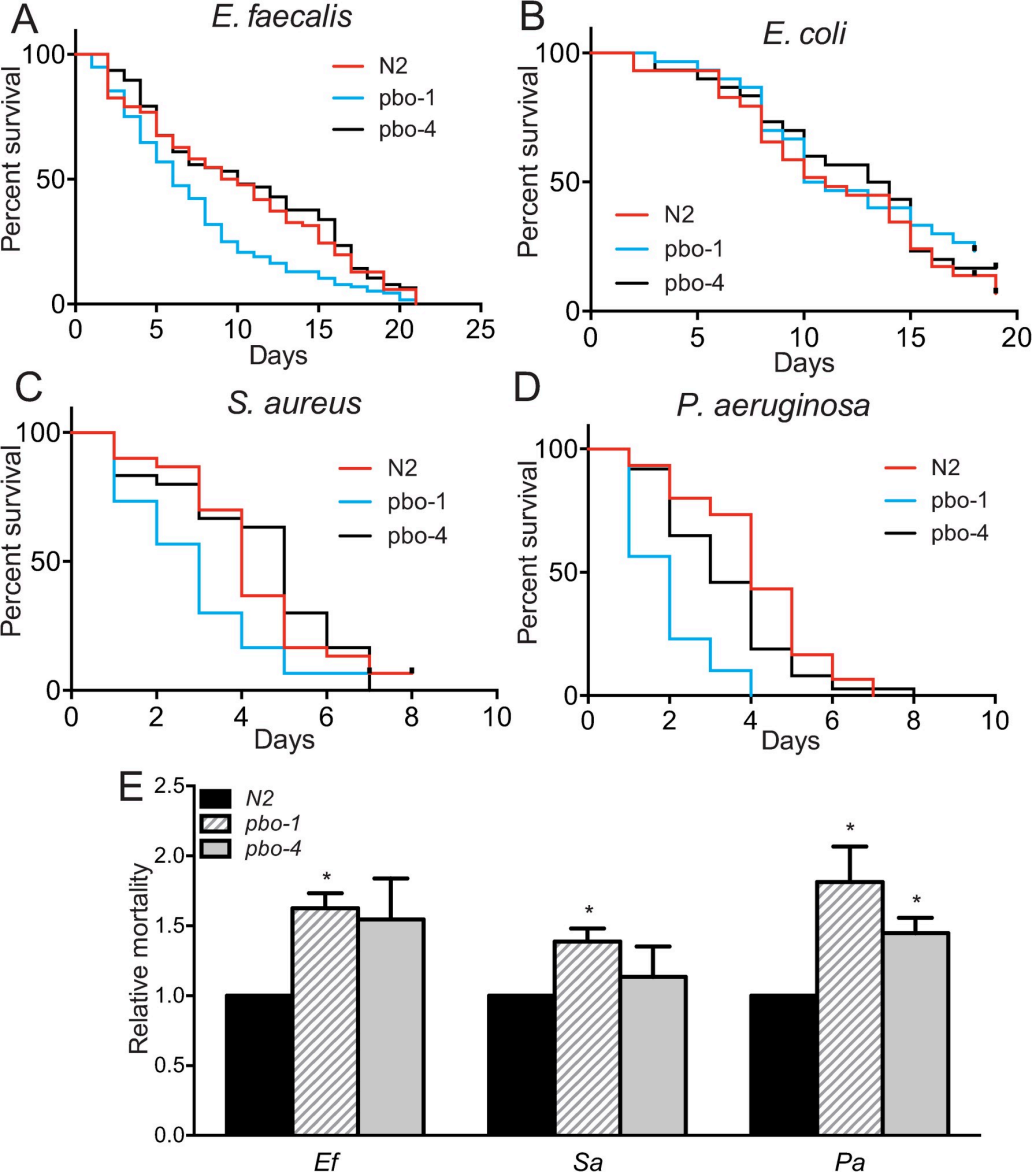
*pbo-1* loss-of-function increases susceptibility to bacterial pathogens. (A-D) Survival or longevity of *C*. *elegans* wild type versus *pbo-1*, or *pbo-4* mutants placed on nematode growth medium (NGM) with a lawn of *E*. *faecalis* (A), *E*. *coli* (B), *S*. *aureus* (C), or *P*. *aeruginosa* (D). In each experiment, 30–100 worms were placed on the pathogen lawn and subsequently transferred every two days to a new NGM plate with a fresh pathogen lawn and monitored for survival. (E) Average relative mortality on pathogen (*E*. *faecalis*, *P*. *aeruginosa* or *S*. *aureus*). Relative mortality is calculated using the lethal time to kill 50% of organisms (LT_50_) and is the ratio of (wild-type LT_50_/mutant LT_50_ on pathogen) over (wild-type LT_50_/mutant LT_50_ on *E*. *coli*). LT_50_ and *p* values for individual experiments used for relative mortality calculations are provided in S1 Table. Relative mortality is shown as the average and standard error of the mean of 3–4 experiments. For each pathogen, statistical analysis by student’s *t* test compared with wild type: *, *p*<0.05.

Our results with KR35 are consistent with defects in proton transport in the intestine leading to the Pbo phenotype, and not the other way around. We hypothesized that the acidic wave may have other physiological consequences to the animals. For example, low intestinal pH could protect the animal from pathogens that entered the alimentary canal during feeding or begin digestion of food deposited in the intestine, analogous to the function of low pH in the stomach of vertebrates. To test this notion, we fed wild type and *pbo-1* mutants the opportunistic pathogen *Enterococcus faecalis* (Fig 2). Similar to previous studies [13, 22], we found that 50% of wild-type animals fed *E*. *faecalis* on nematode growth media (NGM) were killed with a median survival (LT_50_) of 9.5 ± 1 days, post infection (Fig 2A). By contrast, 50% of *pbo-1* mutants were killed after an average of 6 ± 1 days of *E*. *faecalis* feeding, which was significantly shorter than the wild-type animals (*p*<0.005) (Fig 2A). Exposing wild-type *C*. *elegans* to RNAi *pbo-1* prior to *E*. *faecalis* infection also increased susceptibility to *E*. *faecalis* (S2 Fig).

These results indicate that *pbo-1* mutants are more susceptible to killing by *E*. *faecalis* than the wild type. Because *pbo-1* mutants have defects in muscle contraction during defecation, which might slow pathogen clearance, we also tested *pbo-4* mutants. *pbo-4* mutants were not significantly more susceptible than wild type animals with the LT_50_ on average 8 ± 2 days by *E*. *faecalis*. To determine if the increased pathogen sensitivity of *pbo-1* mutants is due to a general defect in lifespan, we grew animals on nonpathogenic *E*. *coli*. The lifespan of wild type, *pbo-1* and *pbo-4 C*. *elegans* mutants were similar in individual experiments (LT_50_ was 12–13 days on average) (Fig 2B).

We also used the *E*. *coli* longevity data to calculate the “relative mortality,” or a measurement of the animal’s survival on pathogens that takes into account the longevity on *E*. *coli* by calculating the ratio of LT_50_ of pathogen-infected animals to that of uninfected animals, with the ratio of wild-type animals normalized to one [23, 24]. The average relative mortality of wild-type animals was significantly less than *pbo-1* mutants (Fig 2E), further supporting that the *pbo-1* mutants are more sensitive to killing by *E*. *faecalis* than the wild type in a manner that is not due to differences in longevity. Thus, our data are consistent with the idea that the *pbo-1* mutant susceptibility to *E*. *faecalis* infections cannot be entirely explained by defects in posterior body contractions during defecation.

To determine if the increased susceptibility of *pbo-1* mutants was limited to *E*. *faecalis*, we tested two other known *C*. *elegans* pathogens, *Pseudomonas aeruginosa* and *Staphylococcus aureus*. In each case, loss of function in *pbo-1* was associated with increased susceptibility, as observed for *E*. *faecalis* (Fig 2). For *S*. *aureus*, survival of the *pbo-4* mutant was not statistically different than wild type (Fig 2), but *pbo-4* survival was slightly reduced on *P*. *aeruginosa* (*p*<0.03), compared to wild type animals on that pathogen.

We also determined the colony-forming-units (CFU) of each pathogen in the intestine of the infected worms to assess whether the susceptibility phenotype of a *pbo-1* mutant was correlated with increased pathogen burden. Worms were exposed to pathogen for 24 or 72 hours, washed to remove surface bacteria, homogenized, and the remaining bacteria were enumerated by plating serial dilutions and counting CFU. Interestingly, there was no increase in the pathogen load of *pbo-1* mutant animals compared with wild type (S3 Fig). In fact, *E*. *faecalis* and *S*. *aureus* were slightly lower in the *pbo-1* mutant. Together, these results indicate that animals lacking *pbo-1* were more susceptible than wild-type to a wide class of pathogens, and the susceptibility phenotype cannot be explained by increased pathogen load in the intestine. The results suggest *pbo-1* might be functioning as part of the innate immune system, responsible for protecting the animal from such pathogens.

### Bicarbonate treatment reverses *pbo-1* susceptibility

Our results indicate *pbo-1* is important for regulating intestinal pH in *C*. *elegans*, consistent with studies reported on the *pbo-1* homolog in vertebrates, including humans [25, 26]. To more directly test whether the abnormally acidic intestinal pH contributes to pathogen susceptibility of the *pbo-1* mutant we carried out infections on plates containing sodium bicarbonate (25 mM, pH = 7). This buffer was chosen because chyme, the acidic and partially digested fluid that leaves the stomach of vertebrates, is buffered by the secretion of bile acids from the gall bladder and bicarbonate from the pancreas as it is transported to the small intestine, thus, bicarbonate is a physiologic buffer for acid in the alimentary canal.

**Fig 3:**
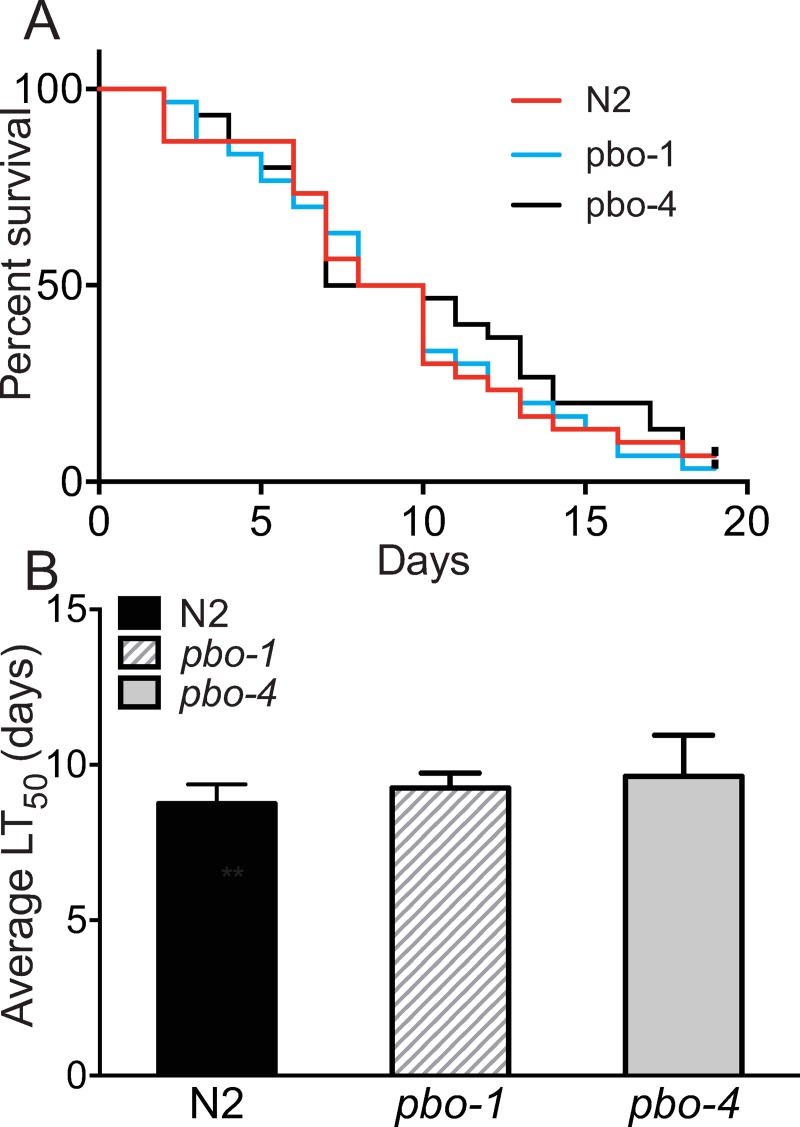
Bicarbonate increases survival of *E*. *faecalis*-fed *pbo-1* mutants. (A) Survival of wild-type, *pbo-1* or *pbo-4* mutants fed *E*. *faecalis* on nematode growth medium with 25 mM bicarbonate (bicarbonate NGM). In each experiment, 30 worms were placed on bicarbonate NGM with an *E*. *faecalis* lawn, and the worms were subsequently transferred every two days to new bicarbonate NGM plates with a fresh *E*. *faecalis* lawn and monitored for survival. (B) Average calculated lethal time to kill 50% of animals (LT_50_) of 3 independent experiments. LT_50_ and *p* values for individual experiments are provided in S2 Table. The average LT_50_ of WT was not statistically different from either mutant by student’s *t*-test (*p*>0.6).

We found that *pbo-1* mutants treated with bicarbonate during *E*. *faecalis* exposure were less susceptible to killing by the pathogen (LT_50_ was 10 ± 2 on 25 mM bicarbonate (Fig 3), compared with 6 ± 1 days with no bicarbonate). Treatment with bicarbonate did not provide wild type or *pbo-4* mutants any additional resistance to *E*. *faecalis* (LT_50_ was 9.5 ± 1 and 9 ± 2 for wild type and *pbo-4*, respectively, on bicarbonate NGM, *p* > 0.25 compared with untreated NGM for each), showing that bicarbonate does not cause general effects on worm susceptibility to pathogens. Thus, we concluded that the increased susceptibility to pathogens was, at least partially, due to the increased acidity of the alimentary canal of *pbo-1* animals. We confirmed that bicarbonate treatment reduced the intestinal acidity with the KR35 dye (S4 Fig). Thus, we concluded that the increased susceptibility to pathogens was, at least partially, due to the increased acidity of the alimentary canal of *pbo-1* animals.

Because intestinal acidity is linked to pathogen susceptibility, and because removing PBO-4 from *pbo-1* mutants reduced the observed acidity of the alimentary canal (Fig 1), we tested the susceptibility of *pbo-1; pbo-4* double mutants to *E*. *faecalis*. These animals were more resistant than the *pbo-1* single mutant, similar to the *pbo-4* single mutant (S5 Fig). Together, our results show that either chemically or genetically reducing acidity in *pbo-1* mutants resulted in increased survival when challenged with pathogens.

### Exposure to pathogens affects the pH in the intestine

**Fig 4:**
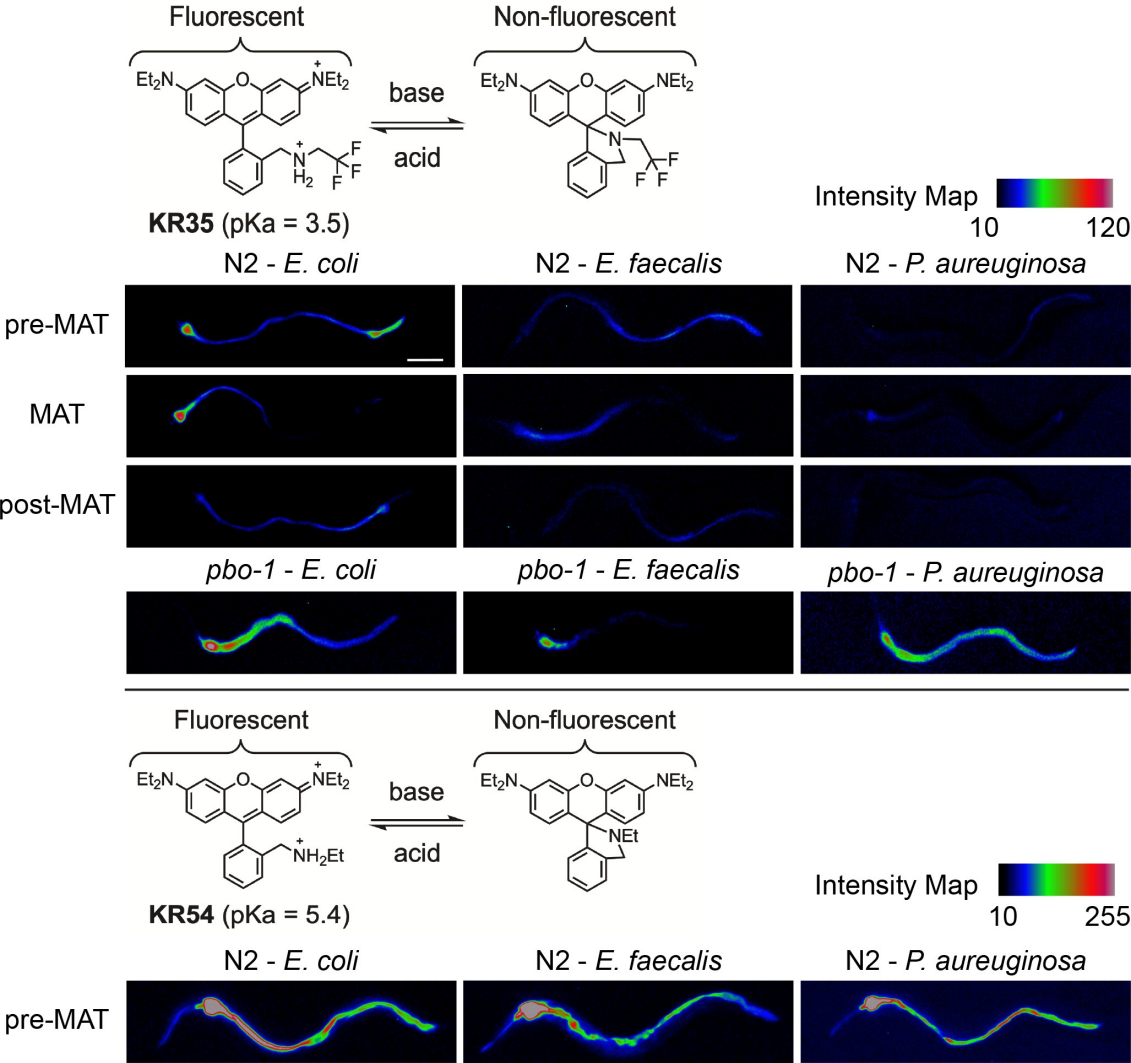
Pathogens alter *C*. *elegans* intestinal pH. The chemical structures of KR35 and KR54 under acidic and basic conditions are shown. The protonated ring-opened form is highly fluorescent, and the deprotonated spirocyclic form is non-fluorescent. Fluorescence micrographs of *C*. *elegans* wild type or *pbo-1* mutant fed *E*. *coli* or pathogen (*E*. *faecalis* or *P*. *aeruginosa*), followed by administration of KR35 (10 μM) for 30 min or KR54 (10 μM) for 10 minutes. Time-dependent images during the DMP extracted from video microscopy are shown. Fluorescence is rendered as a heat map of fluorescence intensity with red representing the most intense fluorescence (high acidity), and black the least intense fluorescence (low acidity) and the map of the intensity values is shown for each fluorophore. The head of the animal is on the left side of each image. (scale bar = 50 μm).

The ability of bicarbonate treatment to suppress pathogen susceptibility in the *pbo-1* mutant suggested that modulation of intestinal pH might be part of the normal response to pathogens. To test this idea, we compared intestinal acidity of the wild-type *C*. *elegans* fed nonpathogenic *E*. *coli* (strain OP50) or a pathogen, either *E*. *faecalis* or *P*. *aeruginosa*, using the KR35 dye. KR35 fluorescence during the MAT appeared to be reduced in animals fed *E*. *faecalis* or *P*. *aeruginosa*, after 24 hours of ingestion, suggesting the intestinal pH was more neutral in pathogen-fed animals (Fig 4, Table 1, S5 and S6 Videos, S6 Fig). We also found a reduction in the number of wild-type animals displaying MATs when fed pathogens, compared to those fed *E*. *coli*, and a change in the inter-MAT interval (Table 1). In wild-type animals fed *E*. *faecalis*, only 11/16 animals displayed MATs, and the interval between cycles was observed as 78 seconds (± 4.6), which was significantly longer than animals fed *E*. *coli* (56 ± 6.1 seconds, P = 0.04).

To rule out that the pathogens were affecting the KR35 dye, and to confirm the neutralization of the intestine, we used KR54, a dye with a closely related chemical structure but a higher pKa of 5.4. Wild-type animals exhibit KR54 fluorescence throughout the intestine, with maximal fluorescence in the anterior, and this pattern was grossly unchanged by feeding either *E*. *faecalis* or *P*. *aeruginosa* (Fig 4). Thus, the change in KR35 fluorescence upon pathogen introduction is more likely to be explained by a neutralizing effect, than a change in the chemical properties of the dye, or other trivial changes in intestinal response to the KR dyes.

We subsequently tested whether pathogen exposure induced pH changes in *pbo-1* mutants. Similar to wild-type animals, the KR35 fluorescence intensity was reduced somewhat in *pbo-1* mutants fed pathogens when compared to *E*. *coli* (Fig 4), and no pH oscillations were observed (S7 and S8 Videos). However, in contrast to wild-type animals, KR35 fluorescence remained increased in *pbo-1* mutants, relative to wild-type animals fed the same pathogen (S6 Fig).

**Fig 5:**
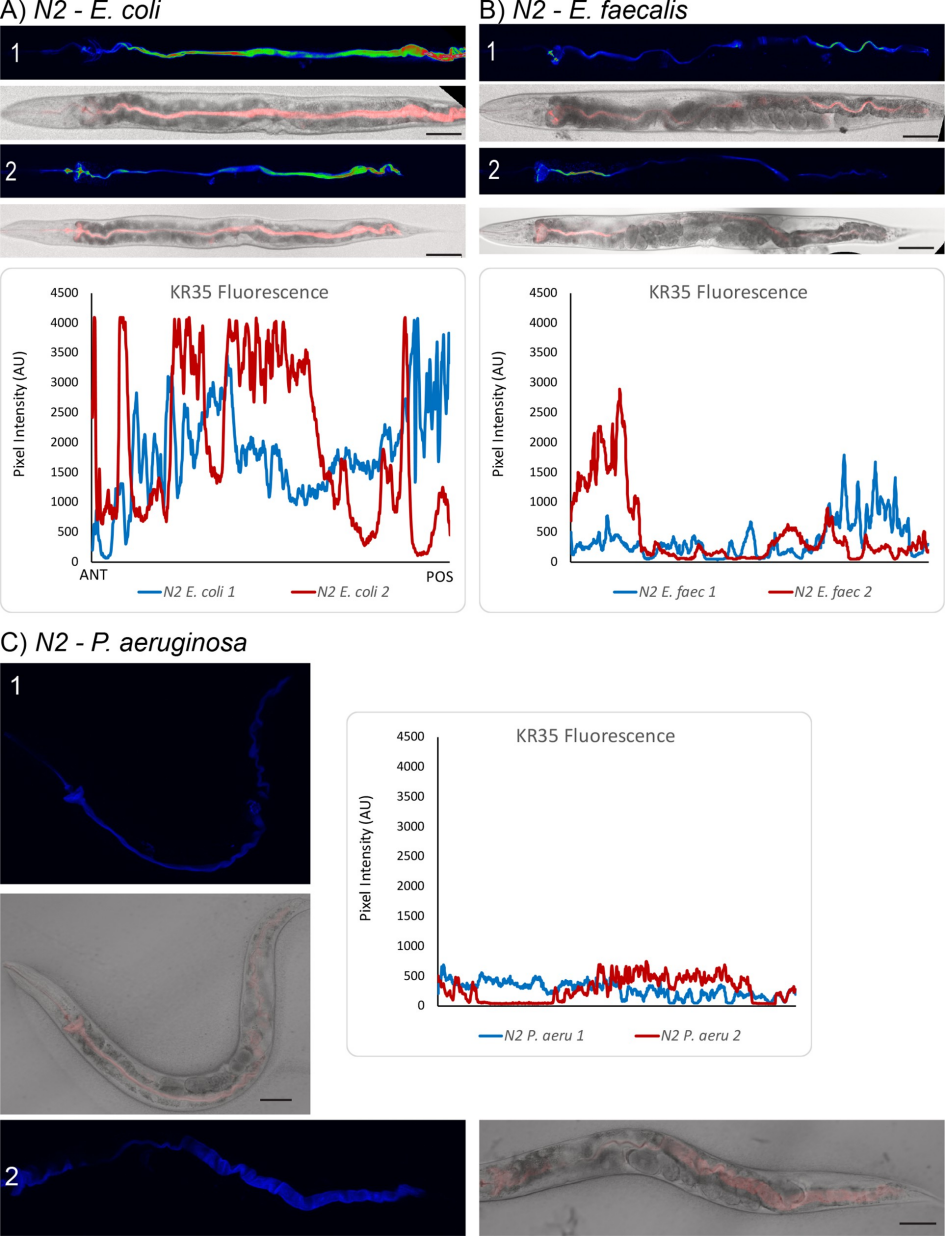
Pathogen ingestion results in intestinal neutralization in wild-type animals. We used laser-scanning confocal microscopy to assess KR35 fluorescence in N2 animals fed *E*. *coli* (A), *E*. *faecalis* (B) or *P*. *aeruginosa* (C). Below the panels for each pathogen is a plot of the pixel intensity of a line drawn from anterior (left) to posterior along the entire intestine. Ingestion of either *E*. *faecalis* or *P*. *aeruginosa* results in reduced KR35 fluorescence, suggesting the pH is more neutral in the intestines of each single animal observed. Scale bars represent 50 μm.

**Fig 6:**
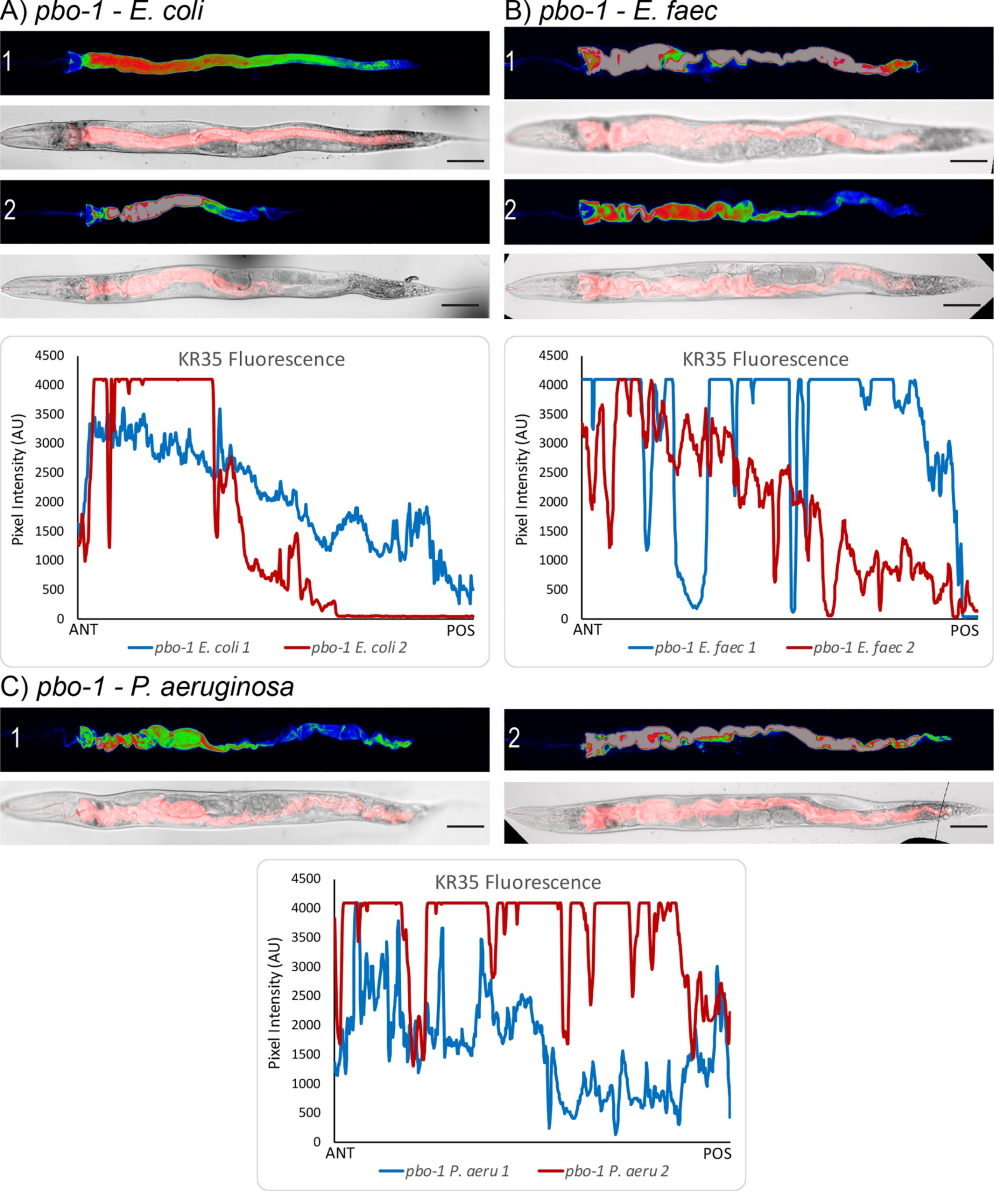
Pathogen ingestion in pbo-1 animals does not change intestinal pH. We used laser-scanning confocal microscopy to assess KR35 fluorescence in multiple *pbo-1(sa7)* animals fed *E*. *coli* (A), *E*. *faecalis* (B) or *P*. *aeruginosa* (C). Below the panels for each pathogen is a plot of the pixel intensity of a line drawn from anterior (left) to posterior along the entire intestine. In these animals we did not observe robust differences in KR35 fluorescence, as a consequence of pathogen ingestion, suggesting intestinal pH change in response to pathogen ingestion partially requires *pbo-1* function. Scale bars represent 50 μm. *pbo-1* mutants exhibit reduced H_2_O_2_ in response to *E*. *faecalis*.

To get higher resolution data on this phenomenon, we used laser-scanning confocal microscopy to visualize KR35 fluorescence in *E*. *coli*, *E*. *faecalis* and *P*. *aeruginosa* fed wild-type (Fig 5) and *pbo-1* mutant animals (Fig 6). For this approach the worms must be immobilized prior to imaging and observations are limited only to the resting/intermediate phase of the DMP (contrasted with the MATs quantified above). Nevertheless, the results confirmed that, in wild type animals, KR35 fluorescence is diminished by ingestion of pathogens. This suggests that part of the normal response to infection is to neutralize the intestinal pH. Conversely, using this method we found fluorescence in *pbo-1* mutants fed pathogens relatively similar to uninfected *pbo-1* mutants. Overall, our results are consistent with pathogen ingestion leading to a more neutral intestinal pH in wild-type animals, and that this change, at least in part, requires *pbo-1* function.

Work from the Ausubel and Garsin labs have shaped our understanding of the molecular events of pathogenesis, and specifically how *E*. *faecalis* and *C*. *elegans* interact during infection [7, 12, 13, 22]. Briefly, recent work has identified that infection of *C*. *elegans* results in the production of H_2_O_2_ as part of the innate immune system [12]. The BLI-3/Duox and SKPO-1 proteins appear to function in the extracellular space of the intestine to produce H_2_O_2_, which has been shown to be protective to the host. Members of the peroxidase family of enzymes have been established to have pH-dependent activity [27, 28]. Thus, we hypothesized that *pbo-1* or infection-dependent changes in pH could regulate the production of H_2_O_2_.

**Fig 7:**
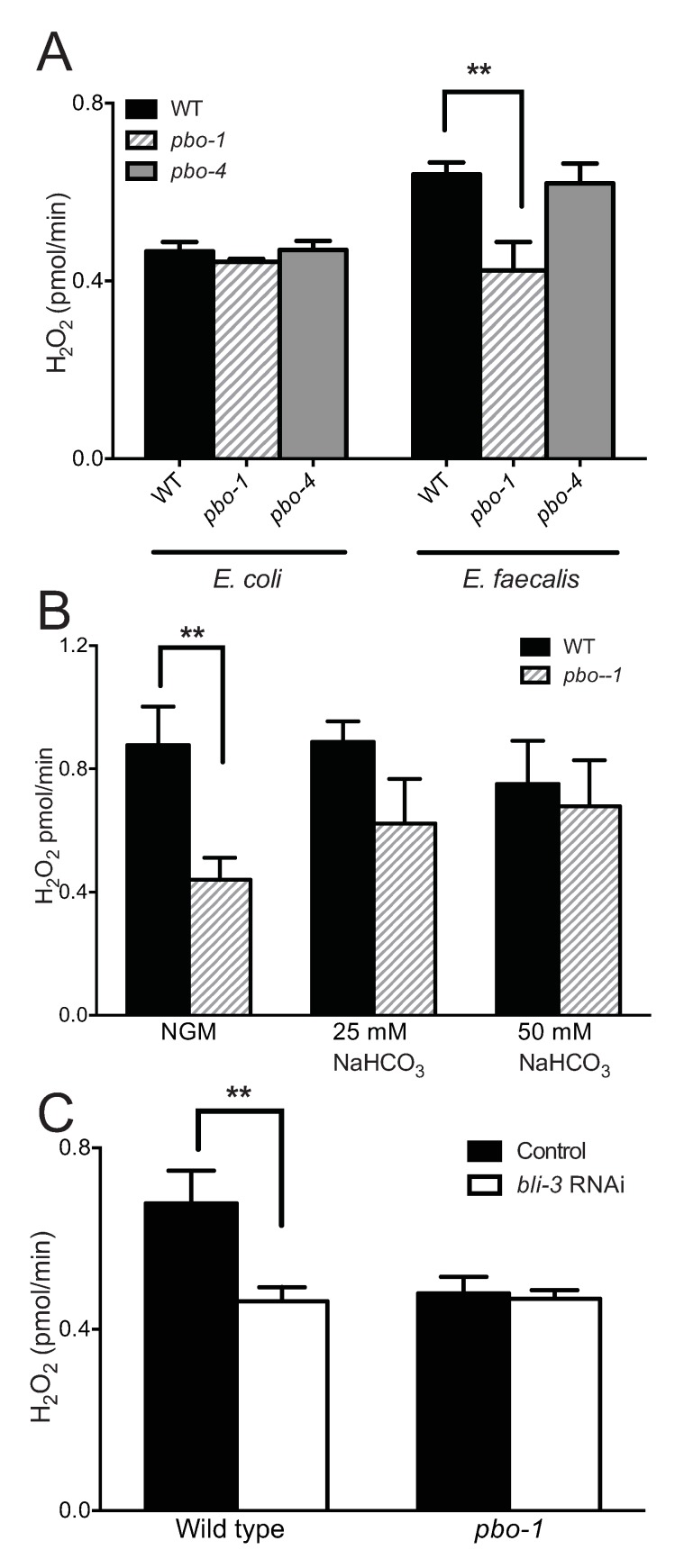
Amplex Red H_2_O_2_ measurements in *E*. *faecalis*-fed worms. (A) PBO-1, but not PBO-4, is important for H_2_O_2_ production in response to *E*. *faecalis*. (B) H_2_O_2_ is restored to a *pbo-1* mutant by adding bicarbonate (NaHCO_3_) to the nematode growth medium. (C) RNAi downregulation of *bli-3* does not further decrease H_2_O_2_ production in *E*. *faecalis-*fed *pbo-1* mutants. Worms were exposed to *bli-3* RNAi *E*. *coli* mixed with the *E*. *coli* empty vector strain at a 1/10 dilution for 3 days prior to feeding with *E*. *faecalis*. Results are of three independent experiments with 30 worms each. Error bars represent standard deviation. **, statistically different by student’s t-test (*p* < 0.01). For part B, wild type and *pbo-1* were not statistically different on NGM + bicarbonate (*p* > 0.4). For part C, *pbo-1* mutants exposed to *bli-3* RNAi were not statistically different from those exposed to the vector control (*p* > 0.5).

The Amplex Red assay (see Materials and Methods) can report on the amounts of H_2_O_2_ produced in response to infection. We used this method to quantify H_2_O_2_ produced by wild-type and *pbo-1* mutant animals infected with *E*. *faecalis* or an *E*. *coli* control. Consistent with previous results [12], the fluorescent product of the Amplex Red assay was increased in animals infected with *E*. *faecalis*, as compared with *E*. *coli*-infected animals (Fig 7A). However, in the *E*. *faecalis-*infected animals, the fluorescent product was reduced in *pbo-1* mutants compared with wild type (Fig 7A). The fluorescent product was similarly reduced in infected animals exposed to RNAi *pbo-1* (S7 Fig). Because bacteria can produce H_2_O_2_ in addition to *C*. *elegans*, to show that the changes in H_2_O_2_ were due to the worm, and not *E*. *faecalis*, we used an *E*. *faecalis* strain deficient for production of H_2_O_2_ (*menB* mutant). As previously reported, animals fed the *E*. *faecalis menB* mutant had levels of Amplex Red fluorescent product that were similar to that of wild-type *E*. *faecalis-*fed animals (S8 Fig). These results support that *pbo-1* mutants are defective for production of H_2_O_2_ and are consistent with the idea that defects in production of H_2_O_2_ in *pbo-1* mutants can account for the increased susceptibility to pathogens.

To test whether H_2_O_2_ production is regulated by changes in pH, we neutralized the intestinal pH of *pbo-1* mutant animals with bicarbonate during infection, then used the Amplex Red assay to quantify H_2_O_2_. Our results showed that the fluorescent product in bicarbonate-treated *pbo-1* mutants was not statistically different from that of wild type (Fig 7B). Thus, bicarbonate reverts the defect in H_2_O_2_ production in *pbo-1* mutants. These results support the conclusion that the reduction of H_2_O_2_ production in the *pbo-1* mutant is pH-dependent.

Because *pbo-4* mutants have an opposite effect on pH in the intestine from that of *pbo-1* mutants, we also tested *pbo-4* mutant for Amplex Red activity. The *pbo-4* mutant showed an Amplex Red phenotype similar to wild type (Fig 7A), suggesting the less acidic pH of the *pbo-4* mutant does not significantly impact the enzymes that produce H_2_O_2_ in our experiment. These results are consistent with the finding that *pbo-4* mutant survival on *E*. *faecalis* and *S*. *aureus* was also not statistically different from wild type (*p*>0.3) (Fig 2). It is notable that there was a statistical difference with *pbo-4* mutant survival on *P*. *aeruginosa* compared with wild type *(p*<0.03). Thus, it is possible that defects in MAT and the acidic wave in the *pbo-4* mutant might cause mild pH-dependent effects on the enzymes that produce H_2_O_2_ or some other effect on pathogen susceptibility in some conditions. Together, the results support the idea that a normally functioning MAT and acidic wave are important for *C*. *elegans* to defend against pathogens via production of H_2_O_2_.

### *bli-3* functions in the same pathway as *pbo-1*

The dual oxidase BLI-3 has been shown to significantly contribute to H_2_O_2_ production in *E*. *faecalis-*fed *C*. *elegans* [12]. We posited that *pbo-1* loss of function might alter H_2_O_2_ production by affecting BLI-3 activity. In this case, downregulating BLI-3 in a *pbo-1* mutant would have no additional effect on H_2_O_2_ production. Standard *bli-3* RNAi results in a severe blistering phenotype and improper development of the worms [12]. Thus, we moderated the downregulation of *bli-3* by exposing worms to a mixture of *bli-3* RNAi bacteria and the vector control (1 to 10 ratio). Exposing worms to this mixture causes a moderate effect on the blistering phenotype and is also known to alter longevity, but it does not significantly alter development [12]. As reported previously [12], we observed a clear defect of H_2_O_2_ production in *bli-3* RNAi treated wild-type worms (Fig 7C). However, there was no additional reduction of H_2_O_2_ production when *pbo-1* mutant animals were similarly exposed to *bli-3* RNAi (Fig 7C). Thus *bli-3* and *pbo-1* appear to function in the same pathway to regulate H_2_O_2_ production in response to *E*. *faecalis*. These results are consistent with the idea that BLI-3 is either directly or indirectly involved in the pH-dependent response to pathogens.

## Discussion

We are interested in characterizing the dynamics of changes in acidity of the *C*. *elegans* intestine and understanding how these changes impact worm physiology. To characterize intestinal acidity, we previously developed a series of acid-activated fluorophores, including KR35 [18], which affords an advantage over other pH-sensitive dyes, *e*.*g*. Oregon Green, that become non-fluorescent under acidic conditions. KR35 has provided a unique view into the dynamic pH changes that occur in the *C*. *elegans* intestine when animals are maintained on non-pathogenic *E*. *coli* [18]. A major finding of the current study is that when we used KR35 to characterize the intestinal pH of animals grown on pathogens, we discovered that the intestine became more alkaline along the length of the lumen, suggesting changes in pH are part of the normal response to infection.

This begs the question as to whether changing the pH is a by-product of the stress response to pathogens or is a critical function itself of innate immunity. We addressed this question using a mutant disrupted in the EF-hand protein, PBO-1. PBO-1 is a critical regulator of ion channels that contribute to the pH of the intestine. Our finding that *pbo-1* mutants are more acidic than wild type in the anterior region of the intestine (Fig 1), and that *pbo-1* mutants are also more susceptible to pathogens (Fig 2) supports the idea that regulation of pH in the intestine is an important component of surviving pathogen infections. Critically, exposing *pbo-1* mutants to bicarbonate, which neutralizes intestinal pH, reverted susceptibility (Fig 3).

Although PBO-1 is also involved in normal defecation [14], the wild-type susceptibly of *pbo-4* mutants and *pbo-1*, *pbo-4* double mutants suggested that the lack of the posterior body contraction during defecation did not lead to the increased pathogen susceptibility (Fig 2). It is important to note that the posterior muscle contraction defects associated with these mutant phenotypes are not necessary for defecation, as these mutants do have expulsion events [14]. Rather, our results support the idea that intestinal pH is linked to protection from pathogens, independent of its role in the defecation motor program.

These results do raise questions about why *pbo-1* deficient animals are more susceptible to pathogens. It is formally possible that aberrantly low pH induces damage or fragility in the epithelial lining of the intestine, and that this vulnerability is exploited by pathogens. Several of our results support that this is not the case. First, the bicarbonate treatment of *pbo-1* mutants rescues the pathogen susceptibility phenotype, supporting the idea that the acidic pH is the primary cause of this phenotype. Second, *pbo-1* mutants have relatively normal longevity on *E*. *coli* (Fig 2), supporting that epithelial integrity of these mutants is grossly normal. Third, our KR35 dye is retained in the lumen and would be fluorescent in the cytoplasm if it were taken up. Our confocal microscopy of KR35-fed animals supports that this dye remains in the lumen (Figs 5 and 6), as does a non-KR dye, Oregon green-labeled dextran (S9 Fig). The observations with these dyes support that the lumen integrity is maintained during the infections. However, there remains a possibility that pathogens might cause damage to an already fragile intestinal lining in these mutants.

Of note, several independent groups have found that pathogens do not need to invade the intestinal cytoplasm to effectively kill *C*. *elegans* [22, 29, 30], and it is unclear if damaging the intestine would increase pathogenicity, although this is quite possible. Of particular interest though is that the Ausubel group has shown that *P*. *aeruginosa* can kill worms by the production of phenazine-derived metabolites, with different products having distinct pH-dependent toxicity [31]. However, phenazines are not produced by *E*. *faecalis* or *S*. *aureus*, and thus would not explain the increased susceptibility of *pbo-1* animals to these bacteria. The observation that *pbo-1* mutants are more vulnerable to three phylogenetically diverse bacterial species does suggest that the consequences of an acidic intestinal pH have broad effects on pathogen susceptibility, rather than targeting a specific virulence mechanism of the bacteria, although this remains to be determined experimentally.

The pH of extracellular spaces has been shown in other contexts to be important for fighting pathogens. In a porcine cystic fibrosis model, the airway surface liquid (ASL) is more acidic than normal animals, and the ASL shows reduced killing of bacterial pathogens [32, 33]. In that model, the authors show that they can also use bicarbonate to simply, and rapidly, restore the antibacterial properties of the ASL [33]. The antimicrobial activity was proposed to be due to pH-modulated antimicrobial peptides (AMPs). We found that in *C*. *elegans* with acidic intestines (*pbo-1* mutant), pH modulated the production of hydrogen peroxide (H_2_O_2_) (Fig 5). H_2_O_2_ has been shown to be an important signaling molecule in physiological contexts such as wound healing and infection. It may be involved in reporting on the presence of pathogens or in the initiation of a protective response. Whether intestinal pH affects the production or stability of H_2_O_2_ remains an open question. Together, these results support that pH modulation of innate immune responses might be conserved across animal classes and systems.

pH also modulates other immune functions. In *in vitro* studies, pH affects the activity of dendritic cells [34], neutrophils [35–37], and the complement system [38]. In patients, lower pH is often associated with reduced immune function [39], and reduced production of bicarbonate is linked with high inflammation [40]. We have shown that in *C*. *elegans*, regulation of intestinal pH appears to be tied to the PBO-1/PBO-4 pathway. These pathways have previously been studied in the context of their role in regulating muscle contraction during defecation. PBO-1 modulates PBO-4 [20], an Na^+^/H^+^ ion exchange pump that normally exports protons from the basolateral membrane of the intestine to adjacent muscles, causing stimulation of the contraction of the posterior body wall muscles prior to defecation [17]. Our results show that PBO-1/PBO-4 might also help to promote host survival through pH-dependent changes in H_2_O_2_ production. It is reasonable to propose that ingestion of pathogens triggers a shift from defecation (muscle contraction) to pathogen defense (production of H_2_O_2_) in *C*. *elegans*. The PBO-1 and PBO-4 proteins are conserved in mammalian cells [15, 16] and it will be interesting to determine whether these proteins have a similar role in regulating pH and immune function in higher species.

## Materials and methods

### Strains and culture conditions

*C*. *elegans* and bacterial strains used in this study are listed in S3 Table. All *C*. *elegans* strains were maintained on nematode growth medium (NGM) at 20°C as previously described [41]) unless otherwise noted. The reference strain for all alleles used and generated was N2 (var. Bristol). All bacterial strains were grown at 37ºC. *E*. *coli* and *P*. *aeruginosa* bacterial strains were grown in low-salt Luria Bertani medium (10 g Bacto-tryptone, 5 g yeast extract, 5 g NaCl), and *E*. *faecalis* and *S*. *aureus* were grown on Brain Heart Infusion, Porcine (BHI, from BD). For *E*. *faecalis*, broth cultures were incubated without shaking. All other bacterial strains were grown with shaking. For selection and expression of RNAi clones, ampicillin (50 **μ**g/mL), tetracycline (15 **μ**g/mL) and isopropyl β-D-1-thiogalactopyranoside (IPTG) (1 mM) were added to the growth medium.

### *C*. *elegans* survival and longevity assays

For survival assays, NGM plates were spread with 10 **μ**L stationary-phase bacteria (*E*. *faecalis*, *S*. *aureus*, or *P*. *aeruginosa* grown to an optical density at 600 nm [OD_600_] ~1), and the plates were incubated overnight at 37°C. Plates were acclimated to room temperature for ~1 hour prior to seeding the plates with *C*. *elegans* (10–30 late larval (L4) staged worms per plate). Worms were scored for survival each day and transferred to new NGM plates with bacterial lawns every two days. Where indicated in the text, NGM was supplemented with 25 mM sodium bicarbonate buffer (pH 7). Longevity assays on *E*. *coli* were conducted as described for survival assays, except NGM plates were spread with 40 **μ**L *E*. *coli* OP50 and incubated overnight at 2°C prior to seeding with *C*. *elegans*.

### RNAi

RNAi was performed by exposing L1-L4-stage larvae to a lawn of *E*. *coli* HT115-expressing dsRNA to target genes on NGM plates for 3 days prior to performing survival or Amplex Red experiments. RNAi clone III-7I12 (*pbo-*1) or clone I-1A17 (*bli-3*) was obtained from the *C*. *elegans* library (Fraser et al. 2000; Kamath et al. 2003).

### Bacterial load during pathogen infection

For pathogen load assays, NGM plates were spread with 40 **μ**L of bacteria (*E*. *coli*, *E*. *faecalis*, *S*. *aureus*, *or P*. *aeruginosa*) and the plates were incubated overnight at 37°C. Plates were acclimated to room temperature prior to seeding with *C*. *elegans* (20 late larval (L4) staged worms per plate). Following 24 or 72-hrs of exposure to pathogen worms were rinsed in 80 **μ**L M9 Buffer, homogenized using a bead disruptor, and serially diluted at 1/10x, 1/100x, 1/1000x, and 1/10000x dilutions. 45 **μ**L of each dilution were spread across Luria Bertani (LB) agar plates and incubated at 37°C overnight. Colony forming units (CFUs) per worm was calculated using the equation E = C x D/P x V/W where E = CFUs per worm, C = number of colonies counted, D = dilution, P = **μ**L plated, V = volume of worm homogenate, and W = number of worms homogenized.

### Amplex Red assay for H_2_O_2_ measurements

The Amplex Red hydrogen peroxide/peroxidase kit (Invitrogen Molecular Probes, Eugene, OR) was used to measure pathogen-stimulated hydrogen peroxide release by *C*. *elegans* as previously described (Chavez et al., 2007, 2009). Briefly, L4 worms were exposed to *E*. *faecalis* for 24 h, and the fluorescence of 10 worms per well was measured after 30 min incubation in the dark with Amplex Red/peroxidase solution (540/590 excitation and emission, respectively). The quantity of H_2_O_2_ was calculated using an etalon curve.

### Fluorophore administration

KR35 and KR54 feeding was carried out as described (Bender et al., 2013), with minor modifications. For non-pathogen videos, animals were reared on NGM plates with OP50, and imaged on plates containing OP50. For pathogen-treated animals, animals were reared to day 1 adults on OP50, then transferred to NGM plates containing either *E*. *faecalis* or *P*. *aeruginosa*, as indicated. Prior to acquisition of videos, young adults were transferred to pathogen-free NGM plates supplemented with 10 **μ**M KR35 for 15–30 minutes or 10 **μ**M KR54 for 5–10 minutes, and then imaged on NGM plates with the indicated bacterial strain. All animals were treated with equivalent conditions on all days of imaging. To minimize any potential variation due to preparation of KR-containing media, the animals of each genotype were fed KR dyes on the same source plate, one condition (genotype or pathogen-fed) at a time, immediately prior to imaging. All animals were removed from the KR-dye plate before the next set of animals were added. At no time were animals of different genotypes mixed together.

### Imaging and image analysis

Videos of free-moving animals fed KR35 and KR54 were acquired on a Leica M165FC microscope using a Leica DFC3000G CCD Camera via the Leica Application Suite software (v 4.40) (Leica Microsystems (Switzerland) Limited). Images sequences were acquired at 5x zoom, with 10x gain, and sequences at 10 frames per second. Illumination was via a Leica Kubler Codix source equipped with an Osram HXP 120W lamp. Images were opened in Fiji (ImageJ), converted to 8-bit, scaled from 0–255 to a dynamic range of 10–120, and a rainbow RGB look up table applied. Movies were converted to AVI using FIJI using an approximate 30 s clip that corresponded to ~10–15 seconds before and after an MAT (where one occurred).

For confocal imaging, wild-type and *pbo-1* were transferred from plates spread with *E*. *coli* (OP50) to plates with either *E*. *faecalis* or *P*. *aeruginosa* for 2–3 hours, then immobilized with 1 mM levamisole and placed on 3% agarose pads and imaged on an Olympus FV1000 laser-scanning confocal microscope. Images were acquired with Fluoview software (ver4.0b). Acquisition conditions were equivalent for all images acquired. Images were opened in ImageJ and the Rainbow RGB look up table applied. Then, a 1-pixel wide line was drawn down the center of the intestine and the Plot Profile command called. The data lists were exported to Excel for graphing. Images were rotated, cropped and/or straightened in ImageJ prior to export to Adobe Illustrator to assemble figures. No intensity scaling was done on any of the images.

For KR35 fluorescence measured by video analysis, animals were reared on *E*. *coli* until the L4 stage, transferred to *E*. *coli*, *E*. *faecalis* or *P*. *aeruginosa* plates and allowed to feed for ~24 hours, with experimenter blind to nematode genotype. Animals were placed on *E*. *coli* plates supplemented with KR35 (10 **μ**m) for 15 minutes. Animals were then transferred to fresh plates and movies acquired for ~3 minutes, to include 3–4 Maximum Anterior Transitions. Movies were opened in ImageJ, and a circular ROI of 25x25 pixels was used to measure the fluorescence of the anterior-most intestine during an MAT. We also measured fluorescence in an adjacent region of the plate, to subtract as background fluorescence. 2–4 measurements were taken per animal, and the data transferred to Excel for graphing and statistical analysis. In animals lacking MATs, we sampled from the anterior-most intestinal region every 45 seconds.

### Statistical analysis

All statistical analyses were carried out using GraphPad Prism version 6.0 (GraphPad Software, San Diego, CA) or Microsoft Excel (Microsoft, Redmond, WA). Student’s paired t-test was used to determine the statistical significance of the Amplex Red data, bacterial burdens, relative mortality and average LT_50._ Statistically significant differences are defined in the figure legends and denoted in the figures with asterisks. Log rank (Mantel-Cox) analysis was used to compare survival and longevity curves pairwise and to calculate the median survival. The median survival and statistical analyses of each of the experiments is provided in S2 and S3 Tables. P-values of <0.05 were considered to be statistically significant.

## Supporting information

S1 Fig MAT Fluorescence compared across genotypes. KR35 fluorescence was quantified from videos of freely moving animals fed *E*. *coli*. Values are the integrated density of a region of interest sampled from the anterior-most segment of the intestine (just posterior to the pharyngeal-intestinal valve), during maximum anterior transitions (MATs). Each point represents the fluorescence measured during a MAT in each genotype. Statistics are P values for comparisons of genotypes to the wild-type (N2) by student’s paired t-test.
(TIF)

S2 Fig Exposure to RNAi *pbo-1* increases susceptibility to *E*. *faecalis*. L1-L4-stage larvae were exposed to a lawn of *E*. *coli* HT115-expressing *pbo-1* dsRNA (clone III-7I12, obtained from the *C*. *elegans* library (Fraser et al. 2000; Kamath et al. 2003)) or HT115 with the vector control for 3 days. 30 worms were subsequently transferred to a lawn of *E*. *faecalis* on nematode growth medium (NGM) to start the survival experiment, then passaged every other day to a fresh *E*. *faecalis* lawn on NGM and monitored for survival. (A) Representative survival experiment. (B) Average calculated lethal time to kill 50% of animals (LT_50_) of 3 independent experiments. Calculated LT_50_ and *p* values for individual experiments are provided in S2 Table. Error bars represent the standard error of the mean. *, statistical significance by *t-*test (*p* < 0.05).
(TIF)

S3 Fig The *pbo-1* mutant does not have increased intestinal bacterial load during infection with pathogens. L4-stage *C*. *elegans* wild type, *pbo-1* or *pbo-4* mutant animals placed on lawns of each pathogen (*E*. *faecalis* (Ef), *S*. *aureus* (Sa), or *P*. *aeruginosa* (Pa) or *E*. *coli* (Ec) and reared at 20°C for 24 (A) or 72 (B) hr, and colony forming units per worm were determined from 5 independent experiments with 20 worms each. Error bars represent the standard error of the mean.
(TIF)

S4 Fig Bicarbonate treatment neutralizes *pbo-1* intestinal pH. *pbo-1* mutants were incubated on NGM plates lacking or supplemented with bicarbonate (25 mM, pH = 7). We observed a reduction in KR35 fluorescence in *pbo-1* mutants in those animals treated with bicarbonate, compared to those not treated. Although the pH was neutralized, none of the *pbo-1* worms observed exhibited any dynamic pH changes.
(TIF)

S5 Fig Survival on *E*. *faecalis* is similar for wild-type and *pbo-1; pbo-4* double mutants. (A) Survival of wild-type, *pbo-1*, *pbo-4*, or *pbo-1; pbo-4* mutants fed *E*. *faecalis* on nematode growth medium (NGM). In each experiment, 30–100 worms were placed on NGM with an *E*. *faecalis* lawn, and the worms were subsequently transferred every two days to new NGM plates with a fresh *E*. *faecalis* lawn and monitored for survival. (B) Average calculated lethal time to kill 50% of animals (LT_50_) of 3 independent experiments. LT_50_ and *p* values for individual experiments are provided in S1 Table. Statistical analysis by student’s *t-*test compared with wild type: *, *p*<0.05. The average LT_50_ of WT was not statistically different from *pbo-4* or *pbo-1*, *4* mutants by student’s *t*-test (*p*>0.15). These data are also partially represented in Fig 2A.
(TIF)

S6 Fig KR35 intensity during MATs after pathogen ingestion. KR35 fluorescence was quantified from videos of freely moving animals fed *E*. *faecalis* or *P*. *aeruginosa*. Values are the integrated density of a region of interest sampled from the anterior-most segment of the intestine (just posterior to the pharyngeal-intestinal valve), during maximum anterior transitions (MATs). Each point represents the fluorescence measured during an MAT in each genotype. Statistics are P values for comparisons of *pbo-1* to the wild-type (N2) within the pathogen treatment, using a student’s t-test. We also compared N2 on *E*. *coli* (S1 Fig) vs. N2 on *E*. *faecalis* and found a significant difference (P = 0.01).
(TIF)

S7 Fig Amplex Red H_2_O_2_ measurements in *pbo-1* RNAi-treated C. elegans. Results are the averages of three independent experiments with 30 worms each performed on three separate days using Amplex Red. L1-L4-stage larvae were exposed to *E*. *coli* HT115 (control) or HT115-expressing dsRNA specific to *pbo-1* for 3 days prior to performing the Amplex Red assay as described in Materials and Methods. Control, worms exposed Error bars represent standard deviation. *, *p*<0.05 by student’s paired t-test.
(TIF)

S8 Fig Amplex Red H_2_O_2_ measurements of the H_2_O_2_-deficient *E*. *faecalis* strain *(ΔmenB*). Results are the averages of at least four independent experiments performed on two separate days. Error bars represent standard deviation. H_2_O_2_ production is not statistically different between WT and ***Δ****menB* for N2 or *pbo-1 C*. *elegans* (*p*>0.1).
(TIF)

S9 Fig Intestinal integrity in *pbo-1* animals. A *pbo-1* mutant fed Oregon Green-labeled dextran (MW 7000) for 30 minutes and then imaged using laser-scanning confocal microscopy. The top image is a z-projected image of the entire animal, the lower panels are single planes through the anterior (left) and posterior (right) regions of the animal. The luminal membrane is marked with a dashed line, while pseudoceolomic membrane is marked with a solid line. The dextran was retained in the intestinal lumen of the animal, and did not appear to diffuse into the cytoplasm. The results suggest the integrity of the epithelial barrier is grossly intact.
(TIF)

S1 Video Wild-type MAT. An N2 animal fed OP50 bacteria supplemented with KR35 exhibiting a single Maximum Anterior Transition (MAT). The video pauses during the MAT, which is indicated by an arrow.
(AVI)

S2 Video *pbo-1* non-MAT. A *pbo-1(sa7)* animal fed OP50 bacteria supplemented with KR35. These animals do not exhibit MATs, but this video captures an equivalent time frame to the wild-type animal in S1 video.
(AVI)

S3 Video *pbo-4* MAT. A *pbo-4(ok583)* mutant animal fed OP50 bacteria supplemented with KR35 exhibiting a single Maximum Anterior Transition (MAT). The video pauses during the MAT, which is indicated by an arrow. The fluorescence intensity is reduced compared to wild-type animals.
(AVI)

S4 Video *pbo-1; pbo-4* MAT. A *pbo-1(sa7); pbo-4(ok583)* double mutant fed OP50 bacteria supplemented with KR35 exhibiting a single Maximum Anterior Transition (MAT). The video pauses during the MAT, which is indicated by an arrow. The fluorescence intensity is reduced compared to wild-type or *pbo-*1 animals.
(AVI)

S5 Video Wild-type MAT on *E*. *faecalis*. An N2 animal fed *E*. *faecalis* supplemented with KR35 exhibiting a single Maximum Anterior Transition (MAT). The video pauses during the MAT, which is indicated by an arrow. The intensity is reduced compared to OP50-fed animals.
(AVI)

S6 Video Wild-type MAT on *P*. *aeuruginosa*. An N2 animal fed *P*. *aeuruginosa* supplemented with KR35 exhibiting a single Maximum Anterior Transition (MAT). The video pauses during the MAT, which is indicated by an arrow. The intensity is reduced compared to OP50-fed animals.
(AVI)

S7 Video *pbo-1* non-MAT on *E*. *faecalis*. A *pbo-1(sa7)* animal fed *E*. *faecalis* supplemented with KR35. These animals do not exhibit MATs, but this video captures an equivalent time frame to the wild-type animal in S5 Video.
(AVI)

S8 Video *pbo-1* non-MAT on *P*. *aeuruginosa*. A *pbo-1(sa7)* animal fed *P*. *aeuruginosa* supplemented with KR35. These animals do not exhibit MATs, but this video captures an equivalent time frame to the wild-type animal in S5 Video.
(AVI)

S1 Table Median survival and p values for pathogen survival and longevity experiments. (DOCX)

S2 Table Median survival and p values for pathogen survival with bicarbonate or RNAi. (DOCX)

S3 Table *C*. *elegans* and bacterial strains used in this study. (DOCX)

S1 Text Supplementary literature cited. (DOCX)
