# Supplementary figures and images for: The C. elegans CHP1 homolog, pbo-1, functions in innate immunity by regulating the pH of the intestinal lumen
Source: PLoS Pathog. 2020 Jan 9;16(1):e1008134. doi: 10.1371/journal.ppat.1008134 (PMC6952083; doi:10.1371/journal.ppat.1008134)

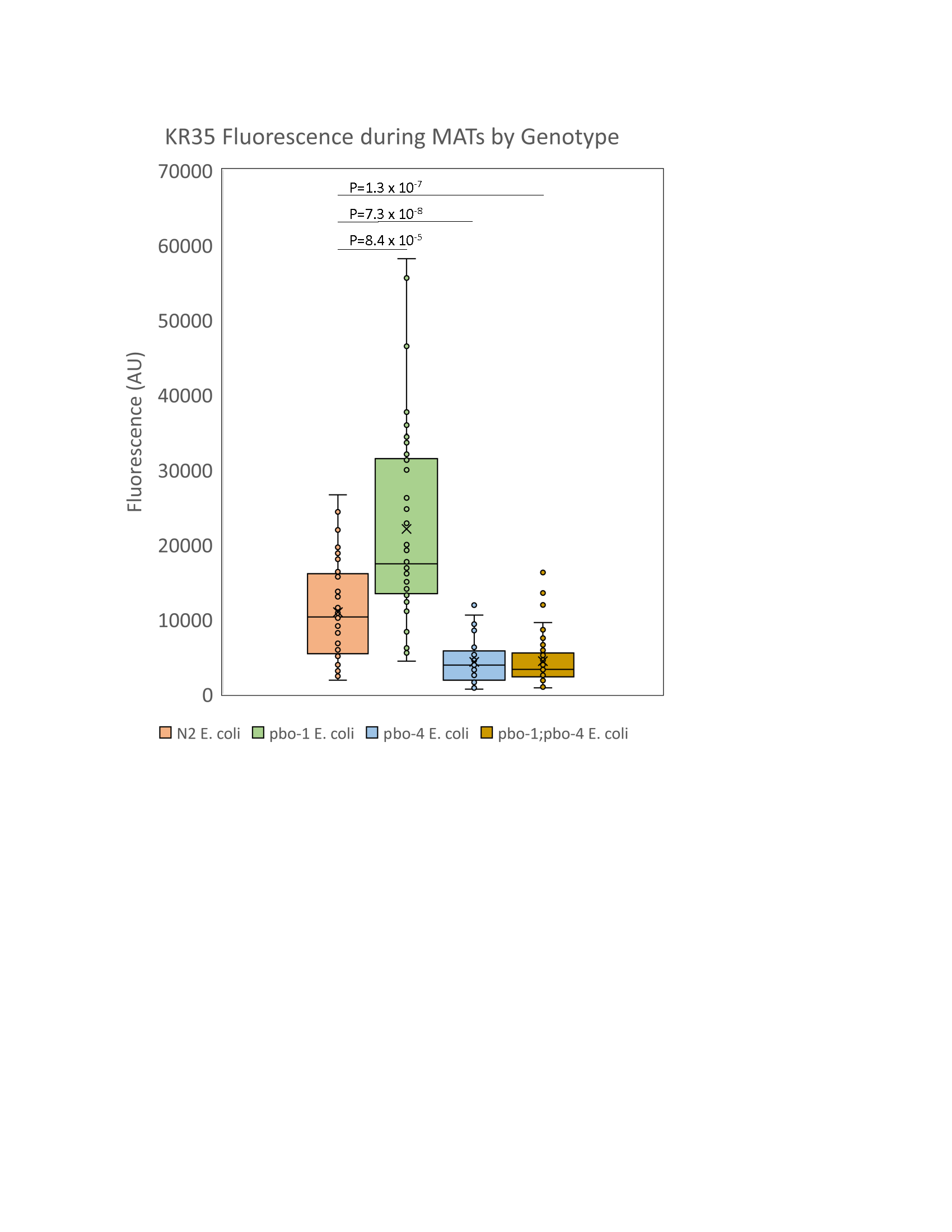

Supplement: S1 Fig — KR35 fluorescence was quantified from videos of freely moving animals fed E. coli. Values are the integrated density of a region of interest sampled from the anterior-most segment of the intestine (just posterior to the pharyngeal-intestinal valve), during maximum anterior transitions (MATs). Each point represents the fluorescence measured during a MAT in each genotype. Statistics are P values for comparisons of genotypes to the wild-type (N2) by student’s paired t-test. (TIF) [file ppat.1008134.s001.tif]

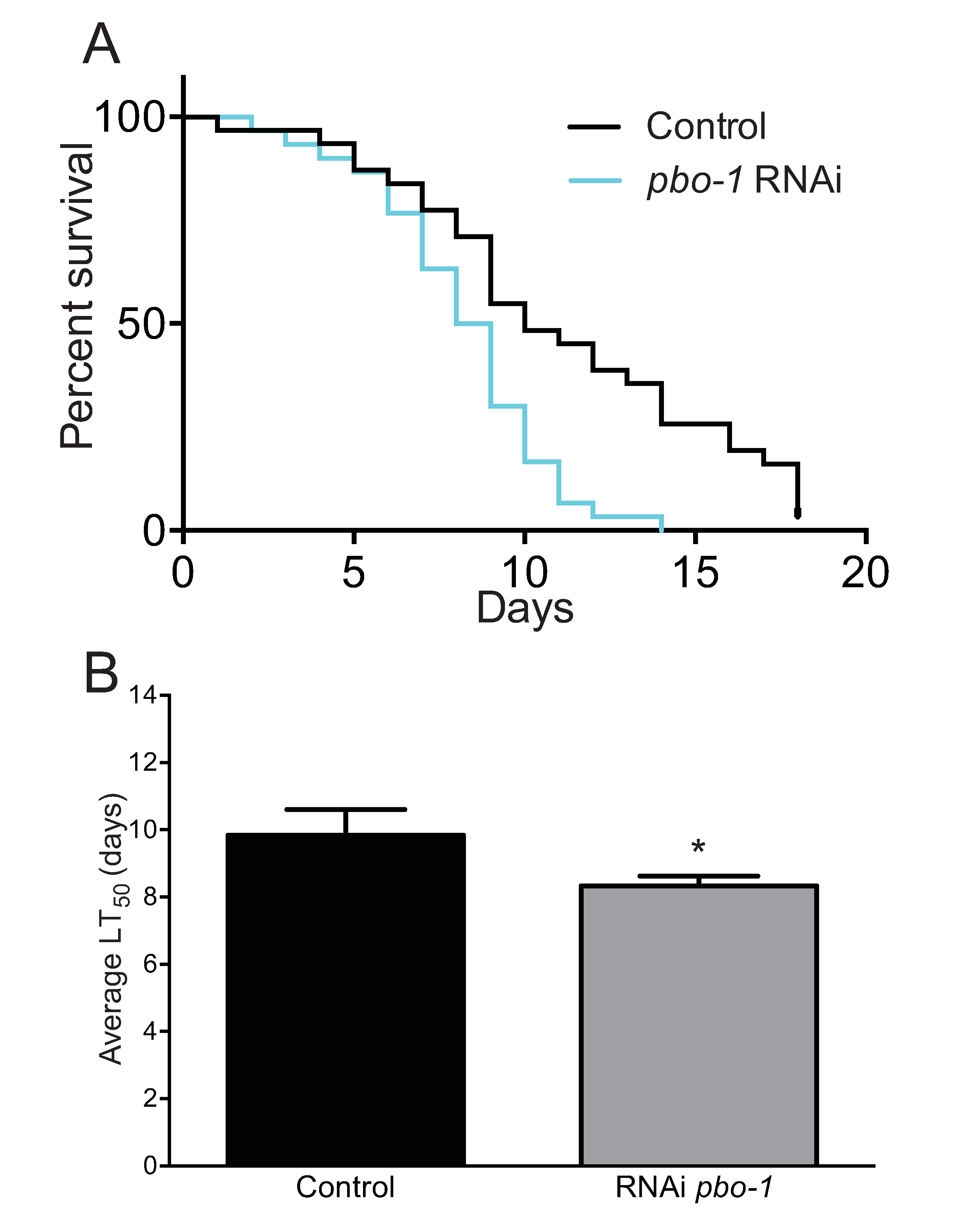

Supplement: S2 Fig — L1-L4-stage larvae were exposed to a lawn of E. coli HT115-expressing pbo-1 dsRNA (clone III-7I12, obtained from the C. elegans library (Fraser et al. 2000; Kamath et al. 2003)) or HT115 with the vector control for 3 days. 30 worms were subsequently transferred to a lawn of E. faecalis on nematode growth medium (NGM) to start the survival experiment, then passaged every other day to a fresh E. faecalis lawn on NGM and monitored for survival. (A) Representative survival experiment. (B) Average calculated lethal time to kill 50% of animals (LT50) of 3 independent experiments. Calculated LT50 and p values for individual experiments are provided in S2 Table. Error bars represent the standard error of the mean. *, statistical significance by t-test (p < 0.05). (TIF) [file ppat.1008134.s002.tif]

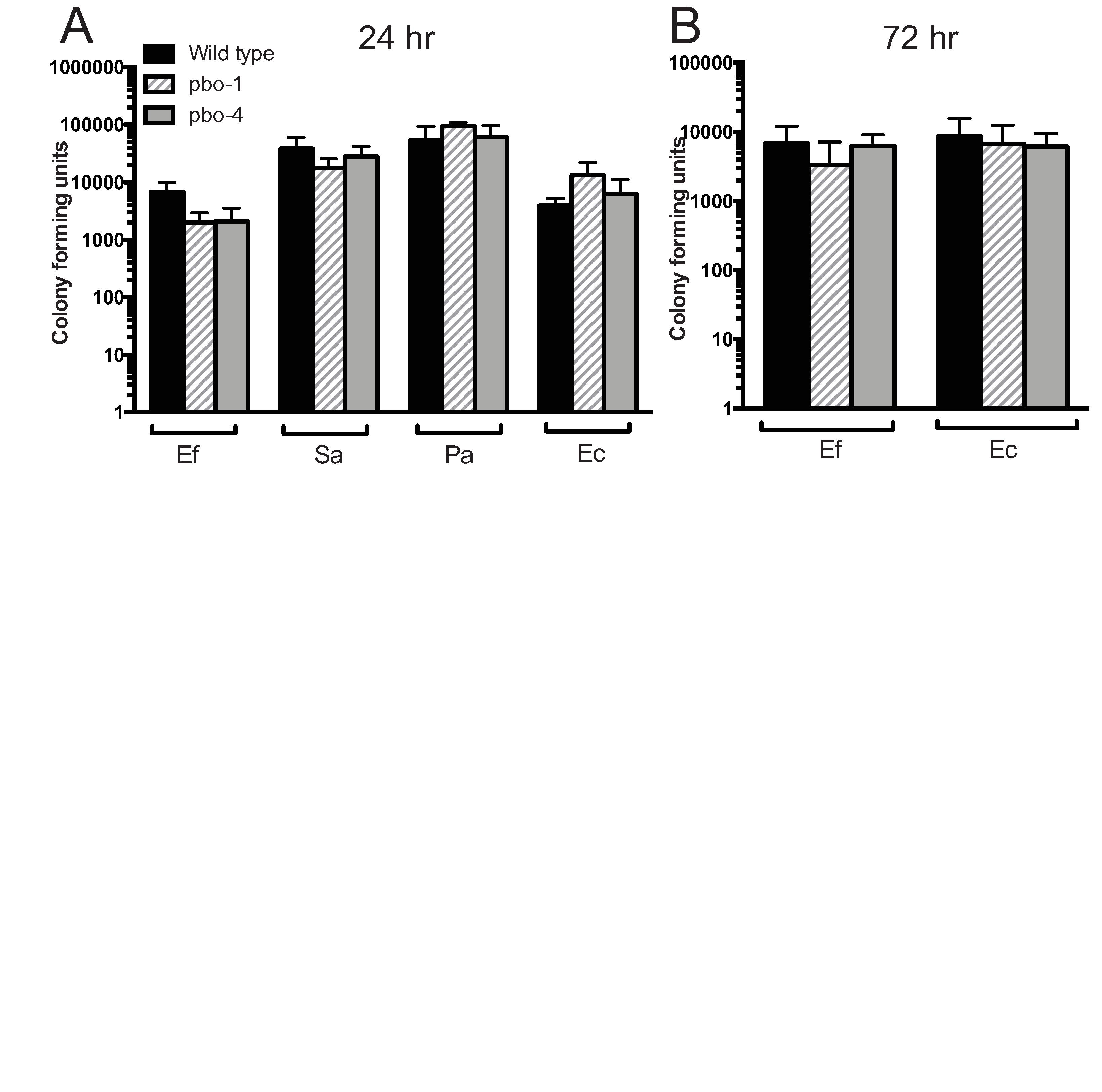

Supplement: S3 Fig — L4-stage C. elegans wild type, pbo-1 or pbo-4 mutant animals placed on lawns of each pathogen (E. faecalis (Ef), S. aureus (Sa), or P. aeruginosa (Pa) or E. coli (Ec) and reared at 20°C for 24 (A) or 72 (B) hr, and colony forming units per worm were determined from 5 independent experiments with 20 worms each. Error bars represent the standard error of the mean. (TIF) [file ppat.1008134.s003.tif]

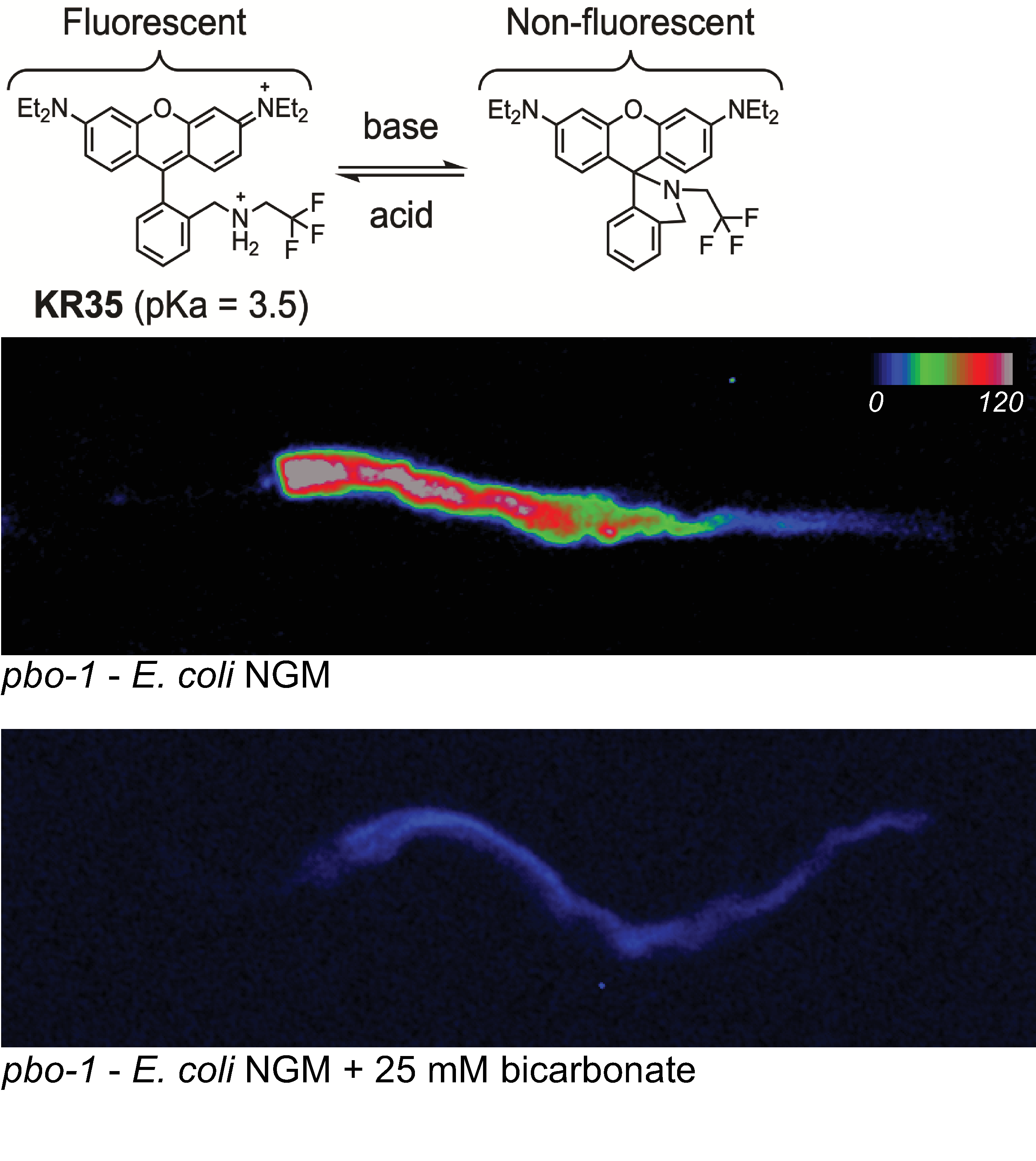

Supplement: S4 Fig — pbo-1 mutants were incubated on NGM plates lacking or supplemented with bicarbonate (25 mM, pH = 7). We observed a reduction in KR35 fluorescence in pbo-1 mutants in those animals treated with bicarbonate, compared to those not treated. Although the pH was neutralized, none of the pbo-1 worms observed exhibited any dynamic pH changes. (TIF) [file ppat.1008134.s004.tif]

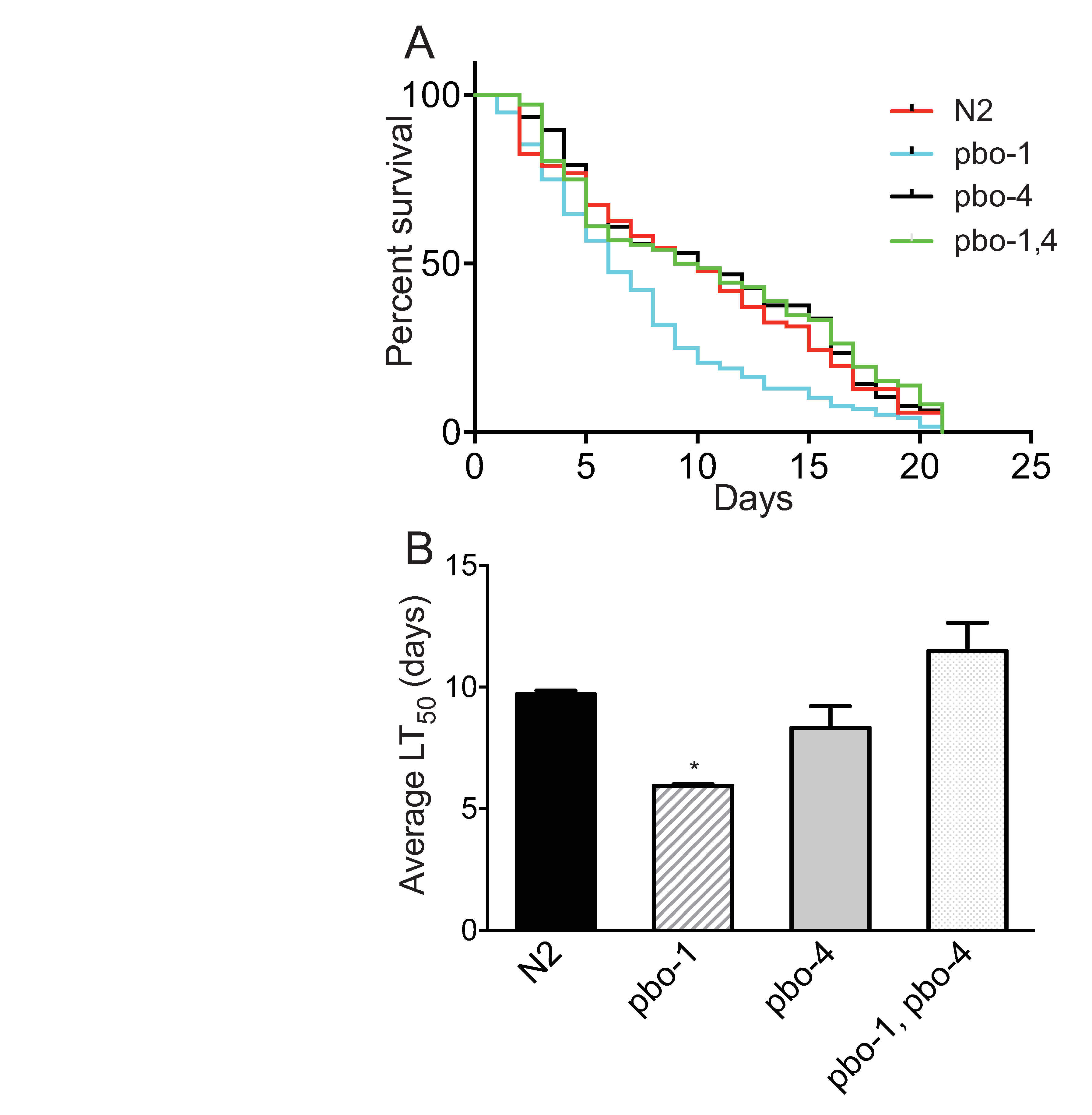

Supplement: S5 Fig — (A) Survival of wild-type, pbo-1, pbo-4, or pbo-1; pbo-4 mutants fed E. faecalis on nematode growth medium (NGM). In each experiment, 30–100 worms were placed on NGM with an E. faecalis lawn, and the worms were subsequently transferred every two days to new NGM plates with a fresh E. faecalis lawn and monitored for survival. (B) Average calculated lethal time to kill 50% of animals (LT50) of 3 independent experiments. LT50 and p values for individual experiments are provided in S1 Table. Statistical analysis by student’s t-test compared with wild type: *, p<0.05. The average LT50 of WT was not statistically different from pbo-4 or pbo-1, 4 mutants by student’s t-test (p>0.15). These data are also partially represented in Fig 2A. (TIF) [file ppat.1008134.s005.tif]

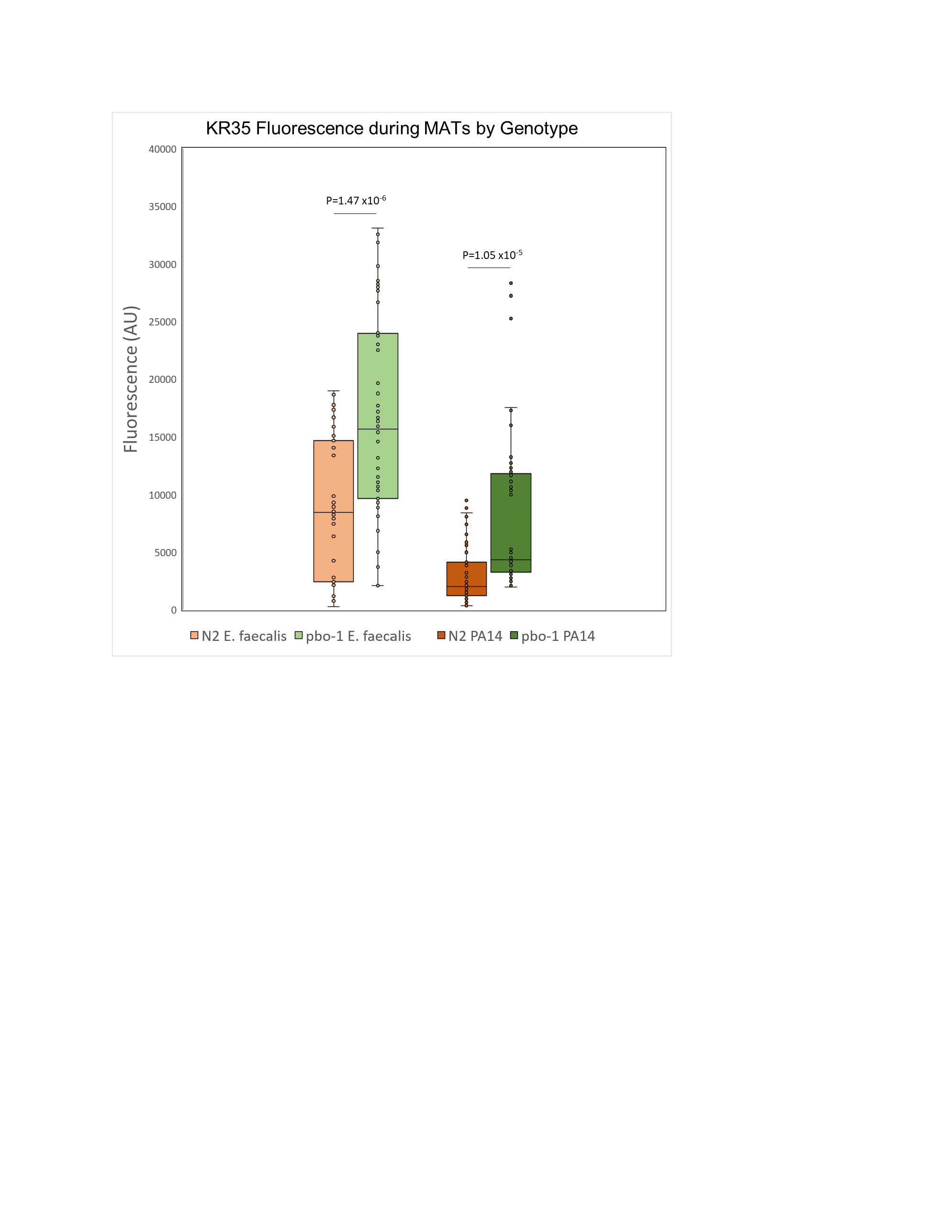

Supplement: S6 Fig — KR35 fluorescence was quantified from videos of freely moving animals fed E. faecalis or P. aeruginosa. Values are the integrated density of a region of interest sampled from the anterior-most segment of the intestine (just posterior to the pharyngeal-intestinal valve), during maximum anterior transitions (MATs). Each point represents the fluorescence measured during an MAT in each genotype. Statistics are P values for comparisons of pbo-1 to the wild-type (N2) within the pathogen treatment, using a student’s t-test. We also compared N2 on E. coli (S1 Fig) vs. N2 on E. faecalis and found a significant difference (P = 0.01). (TIF) [file ppat.1008134.s006.tif]

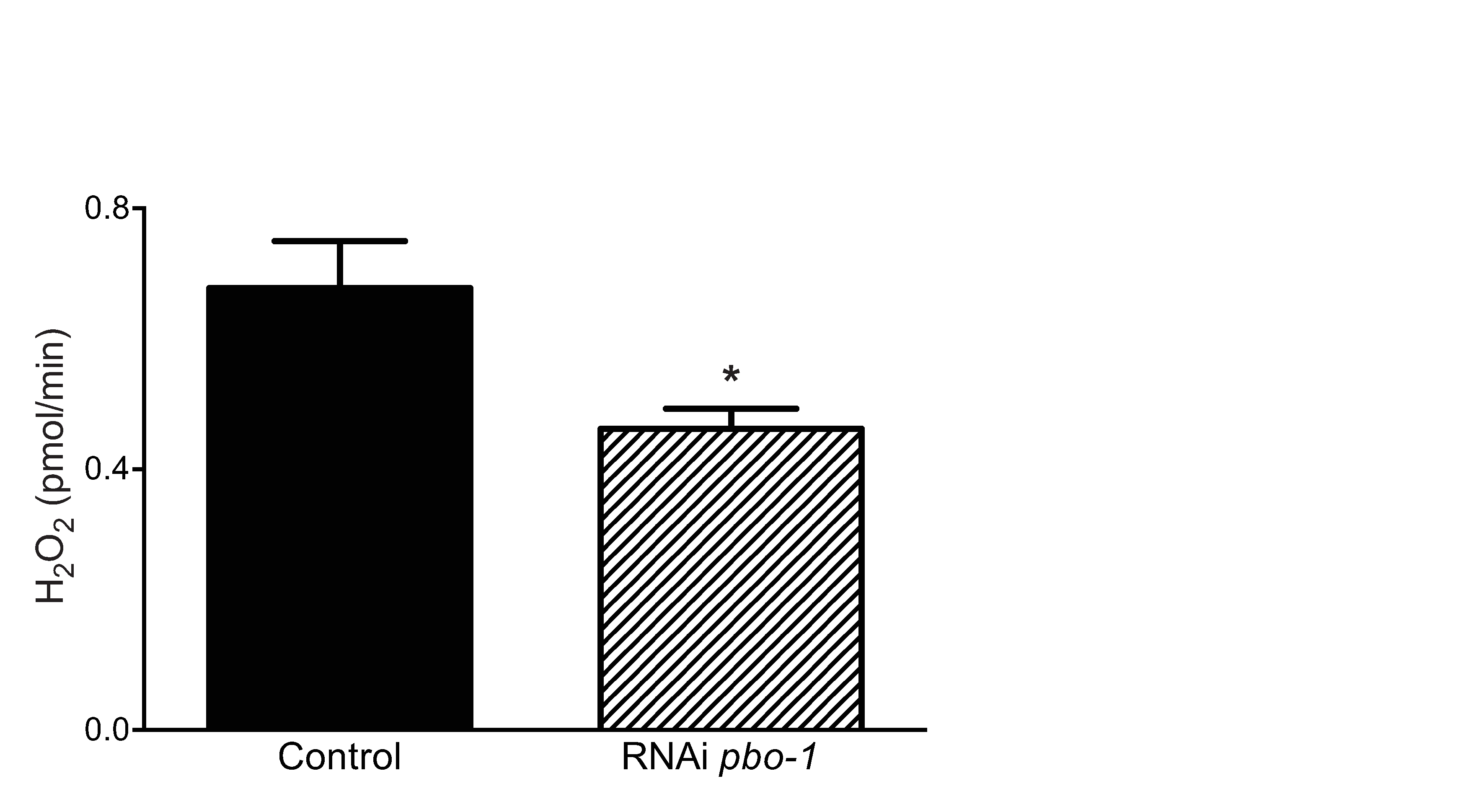

Supplement: S7 Fig — Results are the averages of three independent experiments with 30 worms each performed on three separate days using Amplex Red. L1-L4-stage larvae were exposed to E. coli HT115 (control) or HT115-expressing dsRNA specific to pbo-1 for 3 days prior to performing the Amplex Red assay as described in Materials and Methods. Control, worms exposed Error bars represent standard deviation. *, p<0.05 by student’s paired t-test. (TIF) [file ppat.1008134.s007.tif]

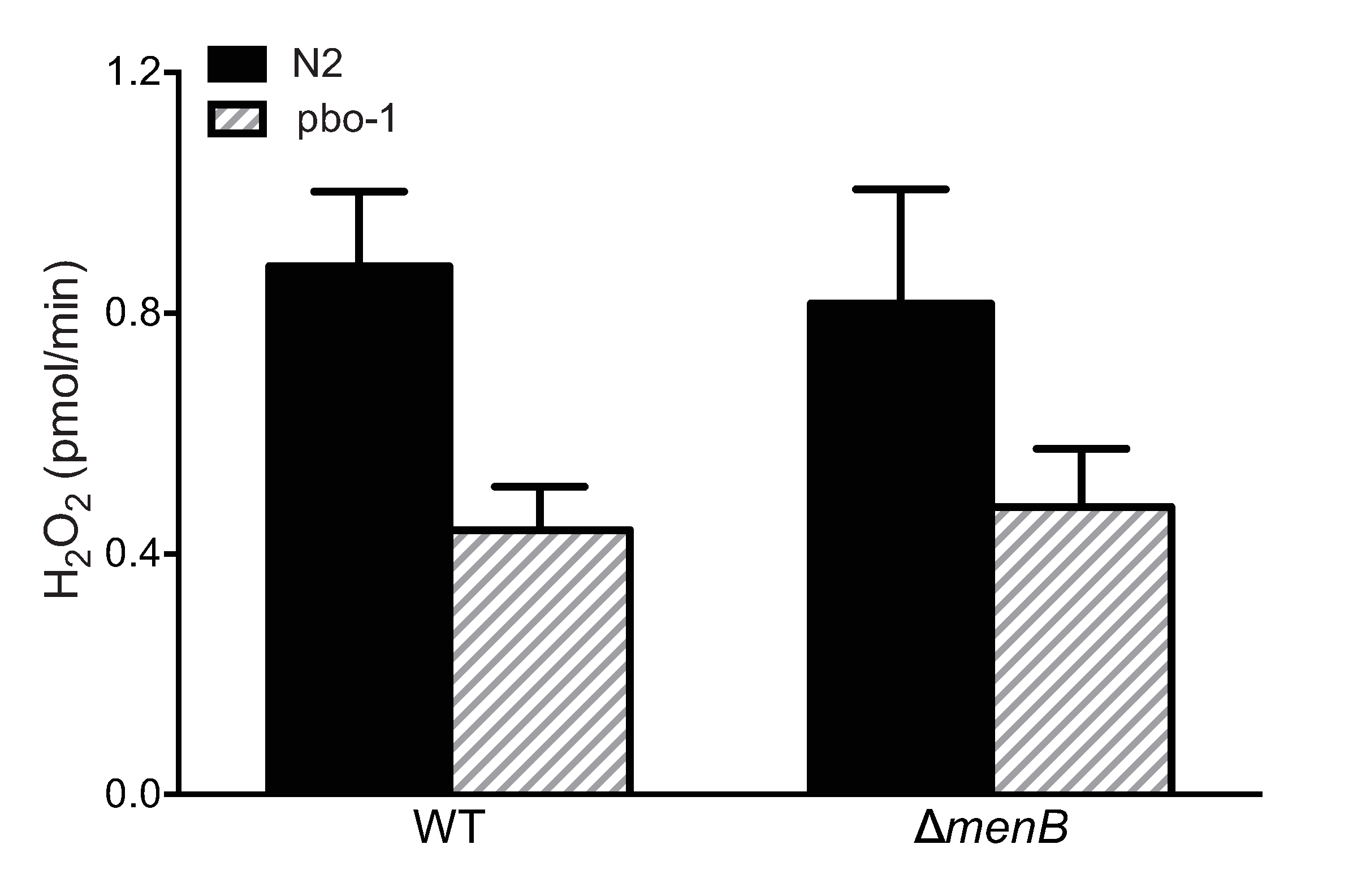

Supplement: S8 Fig — Results are the averages of at least four independent experiments performed on two separate days. Error bars represent standard deviation. H2O2 production is not statistically different between WT and ΔmenB for N2 or pbo-1 C. elegans (p>0.1). (TIF) [file ppat.1008134.s008.tif]

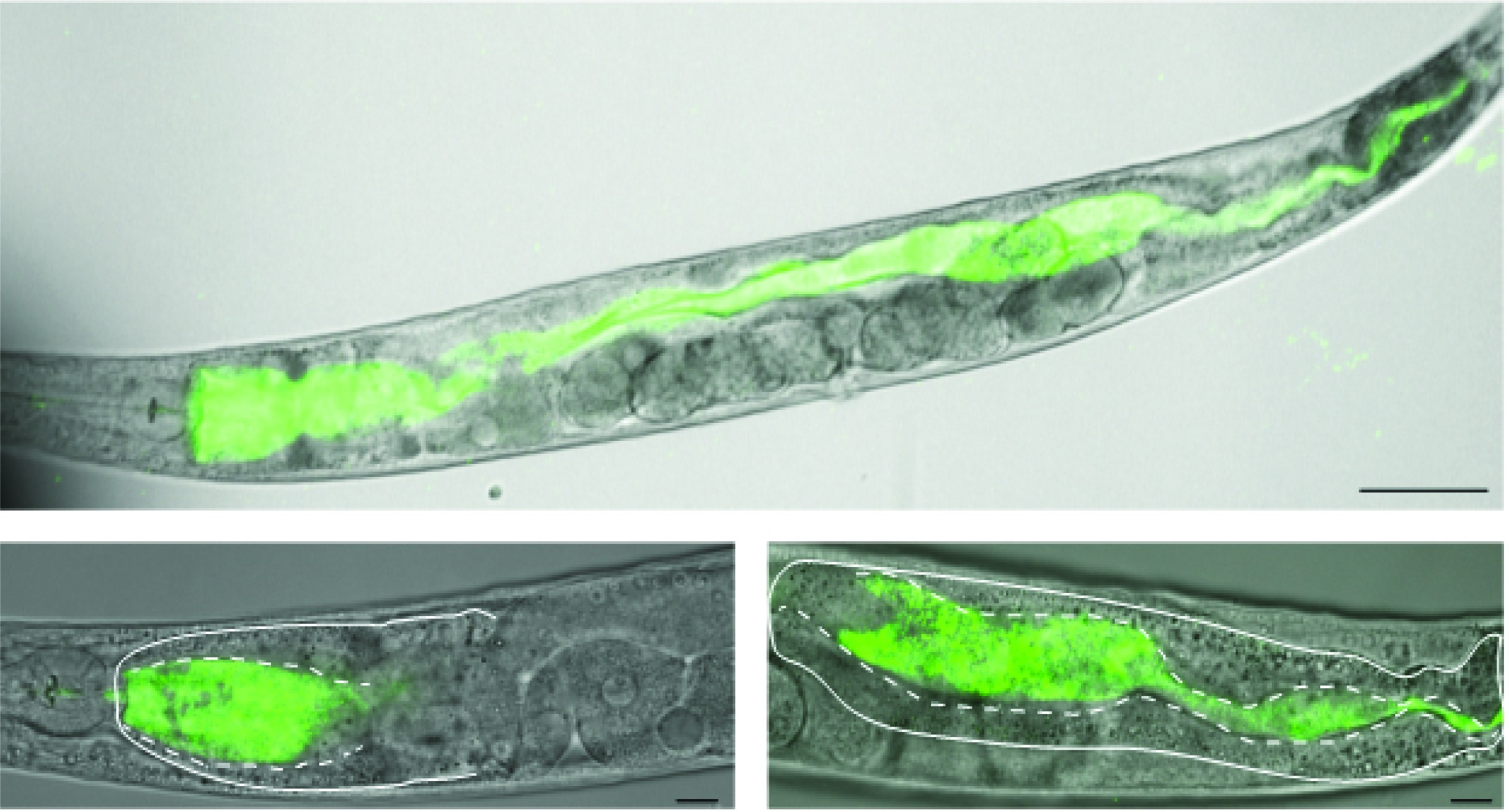

Supplement: S9 Fig — A pbo-1 mutant fed Oregon Green-labeled dextran (MW 7000) for 30 minutes and then imaged using laser-scanning confocal microscopy. The top image is a z-projected image of the entire animal, the lower panels are single planes through the anterior (left) and posterior (right) regions of the animal. The luminal membrane is marked with a dashed line, while pseudoceolomic membrane is marked with a solid line. The dextran was retained in the intestinal lumen of the animal, and did not appear to diffuse into the cytoplasm. The results suggest the integrity of the epithelial barrier is grossly intact. (TIF) [file ppat.1008134.s009.tif]
