# Supplementary material for: The C. elegans CHP1 homolog, pbo-1, functions in innate immunity by regulating the pH of the intestinal lumen
Source: PLoS Pathog. 2020 Jan 9;16(1):e1008134. doi: 10.1371/journal.ppat.1008134 (PMC6952083; doi:10.1371/journal.ppat.1008134)
Supplement: S1 Table — (DOCX) [file ppat.1008134.s018.docx]

| **S1 Table. Median survival and p values for pathogen survival and longevity experiments** | | | |
| --- | --- | --- | --- |
| Experiment | *p value* | Total worms | Median survival (days) |
| *E. faecalis* |  |  |  |
| Exp 1 WT1 | C | 86 | 9.5 |
| vs. pbo-1 | 0.0016 | 116 | 6 |
| vs. pbo-4 | 0.5883 | 77 | 10 |
| vs. pbo-1,4 | 0.3907 | 72 | 9.5 |
| Exp 2 WT | C | 50 | 9 |
| vs. pbo-1 | 0.0008 | 50 | 5 |
| vs. pbo-4 | 0.032 | 40 | 8 |
| vs. pbo-1, 4 | 0.0135 | 40 | 14 |
| Exp 3 WT | C | 80 | 10 |
| vs. pbo-1 | <0.0001 | 80 | 6 |
| vs. pbo-4 | 0.6063 | 70 | 7 |
| vs. pbo-1,4 | 0.0213 | 80 | 11.5 |
|  |  |  |  |
| *E. coli* |  |  |  |
| Exp 1 WT | C | 30 | 11 |
| vs. pbo-1 | 0.4056 | 30 | 10.5 |
| vs. pbo-4 | 0.4535 | 30 | 13.5 |
| vs. pbo-1,4 | 0.3194 | 30 | 12.5 |
| Exp 2 WT | C | 30 | 13 |
| vs. pbo-1 | 0.767 | 30 | 14 |
| vs. pbo-4 | 0.0555 | 30 | 19 |
| vs. pbo-1,4 | 0.0384 | 30 | 21 |
| Exp 3 WT | C | 30 | 15 |
| vs. pbo-1 | 0.1235 | 30 | 13.5 |
| vs. pbo-4 | 0.4778 | 30 | 15 |
| vs. pbo-1,4 | 0.1706 | 30 | 20 |
| Exp 4 WT | C | 30 | 12 |
| vs. pbo-1 | 0.8336 | 30 | 16 |
| vs. pbo-1 | 0.2256 | 30 | 16 |
| vs. pbo-1,4 | 0.5788 | 30 | 14.5 |
|  |  |  |  |
| *P. aeruginosa* |  |  |  |
| Exp 1 WT | C | 30 | 4 |
| vs. pbo-1 | <0.0001 | 40 | 2 |
| vs. pbo-4 | 0.0573 | 40 | 3 |
| Exp 2 WT | C | 30 | 5 |
| vs. pbo-1 | <0.0001 | 30 | 3 |
| vs. pbo-4 | 0.2078 | 30 | 5 |
| Exp 3 WT | C | 30 | 5 |
| vs. pbo-1 | <0.0001 | 30 | 4 |
| vs. pbo-4 | 0.1975 | 30 | 5 |
| Exp 4 WT | C | 30 | 3.5 |
| vs. pbo-1 | 0.0003 | 30 | 2 |
| vs. pbo-4 | 0.8257 | 30 | 3 |
|  |  |  |  |
| *S. aureus* |  |  |  |
| Exp 1 WT | C | 30 | 4 |
| vs. pbo-1 | 0.0248 | 30 | 3 |
| vs. pbo-4 | 0.5348 | 30 | 5 |
| Exp 2 WT | C | 50 | 6 |
| vs. pbo-1 | 0.1743 | 50 | 5 |
| vs. pbo-4 | 0.088 | 50 | 5 |
| Exp 3 WT | C | 50 | 7 |
| vs. pbo-1 | 0.0366 | 50 | 5 |
| vs. pbo-4 | 0.7972 | 50 | 7.5 |
| Exp 4 WT | C | 30 | 5 |
| vs. pbo-1 | 0.281 | 30 | 4 |
| vs. pbo-4 | <0.0001 | 30 | 9 |
