# Supplementary material for: The C. elegans CHP1 homolog, pbo-1, functions in innate immunity by regulating the pH of the intestinal lumen
Source: PLoS Pathog. 2020 Jan 9;16(1):e1008134. doi: 10.1371/journal.ppat.1008134 (PMC6952083; doi:10.1371/journal.ppat.1008134)
Supplement: S2 Table — (DOCX) [file ppat.1008134.s019.docx]

| **S2 Table. Median survival and p values for pathogen survival with bicarbonate or RNAi.** | | | |
| --- | --- | --- | --- |
| Experiment | *p* value | Total worms | Median survival |
|  |  |  |  |
| Bicarbonate |  |  |  |
| Exp 1 WT | C | 30 | 9 |
| vs. pbo-1 | 0.8965 | 30 | 9 |
| vs. pbo-4 | 0.563 | 30 | 9.5 |
| Exp 2 WT | C | 30 | 10 |
| vs. pbo-1 | 0.3185 | 30 | 10 |
| vs. pbo-4 | 0.5861 | 30 | 11 |
| Exp 3 WT | C | 30 | 7 |
| vs. pbo-1 | 0.5509 | 30 | 10 |
| vs. pbo-4 | 0.321 | 30 | 12.5 |
| Exp 4 WT | C | 30 | 9 |
| vs. pbo-1 | 0.6379 | 30 | 8 |
| vs. pbo-4 | 0.3531 | 30 | 6.5 |
|  |  |  |  |
| RNAi |  |  |  |
| Exp 1 |  |  |  |
| Vector | C | 30 | 10 |
| *pbo-1* RNAi | 0.0009 | 30 | 8.5 |
| Exp 2 |  |  |  |
| Vector | C | 30 | 9 |
| *pbo-1* RNAi | 0.0105 | 30 | 8 |
| Exp 3 |  |  |  |
| Vector | C | 30 | 10.5 |
| *pbo-1* RNAi | 0.0028 | 30 | 8.5 |
