# Supplementary material for: The C. elegans CHP1 homolog, pbo-1, functions in innate immunity by regulating the pH of the intestinal lumen
Source: PLoS Pathog. 2020 Jan 9;16(1):e1008134. doi: 10.1371/journal.ppat.1008134 (PMC6952083; doi:10.1371/journal.ppat.1008134)
Supplement: S1 Text — (DOCX) [file ppat.1008134.s021.docx]

# Supplemental Material Literature Cited

1. Brenner S. The genetics of *Caenorhabditis elegans*. Genetics. 1974;77(1):71-94. PubMed PMID: 4366476; PubMed Central PMCID: PMCPMC1213120.

2. Pfeiffer J, Johnson D, Nehrke K. Oscillatory transepithelial H(+) flux regulates a rhythmic behavior in *C. elegans*. Current biology : CB. 2008;18(4):297-302. Epub 2008/02/23. doi: 10.1016/j.cub.2008.01.054. PubMed PMID: 18291648; PubMed Central PMCID: PMCPMC2350219.

3. Thomas JH. Genetic analysis of defecation in *Caenorhabditis elegans*. Genetics. 1990;124(4):855-72. Epub 1990/04/01. PubMed PMID: 2323555; PubMed Central PMCID: PMCPMC1203977.

4. Fraser AG, Kamath RS, Zipperlen P, Martinez-Campos M, Sohrmann M, Ahringer J. Functional genomic analysis of C. elegans chromosome I by systematic RNA interference. Nature. 2000;408(6810):325-30. Epub 2000/12/01. doi: 10.1038/35042517. PubMed PMID: 11099033.

5. Kamath S, Kapatral V, Chakrabarty AM. Cellular function of elastase in Pseudomonas aeruginosa: role in the cleavage of nucleoside diphosphate kinase and in alginate synthesis. Molecular microbiology. 1998;30(5):933-41. PubMed PMID: 9988471.

6. Dunny GM, Brown BL, Clewell DB. Induced cell aggregation and mating in Streptococcus faecalis: evidence for a bacterial sex pheromone. Proceedings of the National Academy of Sciences of the United States of America. 1978;75(7):3479-83. PubMed PMID: 98769; PubMed Central PMCID: PMCPMC392801.

7. Rahme LG, Stevens EJ, Wolfort SF, Shao J, Tompkins RG, Ausubel FM. Common virulence factors for bacterial pathogenicity in plants and animals. Science. 1995;268(5219):1899-902. PubMed PMID: 7604262.

8. Duthie ES, Lorenz LL. Staphylococcal coagulase; mode of action and antigenicity. J Gen Microbiol. 1952;6(1-2):95-107. doi: 10.1099/00221287-6-1-2-95. PubMed PMID: 14927856.
